# Th17 cytokine differentiation and loss of plasticity after SOCS1 inactivation in a cutaneous T-cell lymphoma

**DOI:** 10.18632/oncotarget.9077

**Published:** 2016-04-28

**Authors:** Stefan Ehrentraut, Björn Schneider, Stefan Nagel, Claudia Pommerenke, Hilmar Quentmeier, Robert Geffers, Maren Feist, Maren Kaufmann, Corinna Meyer, Marshall E. Kadin, Hans G. Drexler, Roderick A. F. MacLeod

**Affiliations:** ^1^ Leibniz Institute - DSMZ, German Collection of Microorganisms and Cell Cultures, Department of Human and Animal Cell Cultures, Braunschweig, Germany; ^2^ University of Rostock, Institute of Pathology and Molecular Pathology, Rostock, Germany; ^3^ HZI - Helmholtz Center for Infection Research, Genome Analytics Research Group, Braunschweig, Germany; ^4^ University Medical Center Goettingen, Department of Haematology and Medical Oncology, Goettingen, Germany; ^5^ Department of Dermatology and Skin Surgery, Roger Williams Medical Center, Boston University School of Medicine, Providence, RI, USA

**Keywords:** CTCL, IL-2, IL-17F, JAK3, SOCS1

## Abstract

We propose that deregulated T-helper-cell (Th) signaling underlies evolving Th17 cytokine expression seen during progression of cutaneous T-cell lymphoma (CTCL). Accordingly, we developed a lymphoma progression model comprising cell lines established at indolent (MAC-1) and aggressive (MAC-2A) CTCL stages. We discovered activating JAK3 (V722I) mutations present at indolent disease, reinforced in aggressive disease by novel compound heterozygous SOCS1 (G78R/D105N) JAK-binding domain inactivating mutations. Though isogenic, indolent and aggressive-stage cell lines had diverged phenotypically, the latter expressing multiple Th17 related cytokines, the former a narrower profile. Importantly, indolent stage cells remained poised for Th17 cytokine expression, readily inducible by treatment with IL-2 - a cytokine which mitigates Th17 differentiation in mice. In indolent stage cells JAK3 expression was boosted by IL-2 treatment. Th17 conversion of MAC-1 cells by IL-2 was blocked by pharmacological inhibition of JAK3 or STAT5, implicating IL2RG - JAK3 – STAT5 signaling in plasticity responses. Like IL-2 treatment, SOCS1 knockdown drove indolent stage cells to mimic key aggressive stage properties, notably IL17F upregulation. Co-immunoprecipitation experiments showed that SOCS1 mutations abolished JAK3 binding, revealing a key role for SOCS1 in regulating JAK3/STAT5 signaling. Collectively, our results show how JAK/STAT pathway mutations contribute to disease progression in CTCL cells by potentiating inflammatory cytokine signaling, widening the potential therapeutic target range for this intractable entity. MAC-1/2A cells also provide a candidate human Th17 laboratory model for identifying potentally actionable CTCL markers or targets and testing their druggability *in vitro*.

## INTRODUCTION

Cutaneous T-cell lymphomas (CTCL) form a diverse group of skin neoplasms, including (premalignant) lymphomatoid papulosis (LyP), malignant anaplastic large cell lymphoma and mycosis fungoides, together with the systemic, aggressive Sézary syndrome [1]. The molecular pathological relationships between these entities remain obscure, while the transition between self-healing LyP and malignant CTCL presents an intriguing model for tumor progression and the emerging roles of epigenetics and neoplastic stem cells. Reflecting their enigmatic and complex molecular pathology, the search for curative treatments for CTCL has proved challenging.

Like those observed in non-malignant T-helper cells, cytokine profiles of malignant cells in CTCL depend on stimuli they receive from their environment, and phenotypic changes acquired during disease progression invite comparisons with physiological T-cell development [2]. While early CTCL may present T-reg (ulatory cell) features, progression may be accompanied by the acquisition of T-h(elper cell)-17 properties [2]. Recently, it was shown that IL-17 expression and secretion is a key feature in subsets of patients with chronic dermatitis or CTCL [3, 4], supporting an integral role for Th17 differentiation in CTCL progression [2].

JAK3 is a tyrosine kinase associated with common gamma-chain receptors of cytokine signaling, thereby preferentially phosphorylating and activating STAT5 [5], a pathway which supports IFN-gamma expression during Th1 cell development [6]. JAK3 mutations have already been described in megakaryoblastic, T-cell prolymphocytic, natural killer T-cell, and T-cell acute lymphoblastic leukemias [5, 7–10]. These studies revealed several activating mutations affecting the pseudokinase domain of JAK3 which abolish cytokine dependence in Ba/F3 and MOHITO cell lines [9, 10].

SOCS proteins constitute a group of negative feedback regulators of JAK/STAT signaling [11]. The function of SOCS1 is neatly illustrated by its aliases: (1) JAB for JAK kinase binding protein [12], (2) SSI1 for SH2 domain containing STAT interacting protein [13], and (3) suppressor of cytokine signaling for its ability to block IL-6 mediated T-cell differentiation [14]. Indeed, SOCS1 binds and inhibits the JAK kinase activation loop via the SH2 domain [15]. Mutations in SOCS1 have previously been shown to stabilize p(hospho-) JAK2 in primary mediastinal B- cell lymphoma [16] and boost pSTAT5 stockpiling in Hodgkin lymphoma [17]. Hitherto, direct links between SOCS1 and JAK3 have remained tenuous.

Human CTCL derived cell lines provide infinitely verifiable *in vitro* platforms for investigating both the molecular pathology and the druggability of therapeutic targets identified therein or clinically. MAC-1 and MAC-2A cell lines were respectively established at indolent and aggressive disease phases from a cutaneous lymphoma patient initially presenting with LyP [18, 19]. To parse genomic alterations discovered therein we performed whole exome sequencing combined with cytogenetic and genomic array analyses. Here we probe the impact of a novel set of SOCS1 mutations acquired at disease progression on JAK/STAT signaling constitutively activated by JAK3 mutation. Significantly, SOCS1 knockdown performed on indolent phase MAC-1 cells induced inflammatory Th17-related cytokines seen at tumor progression, thus mimicking the effects of exogenous IL-2. Taken together, our findings endorse an isogenic cell line resource for identifying new therapeutic targets in T-cell lymphoma and a model for testing druggability responses thereof, while pinpointing a further role for disordered JAK-STAT signaling in malignant T-cell differentiation.

## RESULTS

### Isogenic MAC cell lines carry earlier JAK3 and later SOCS1 mutations

**Figure 1:**
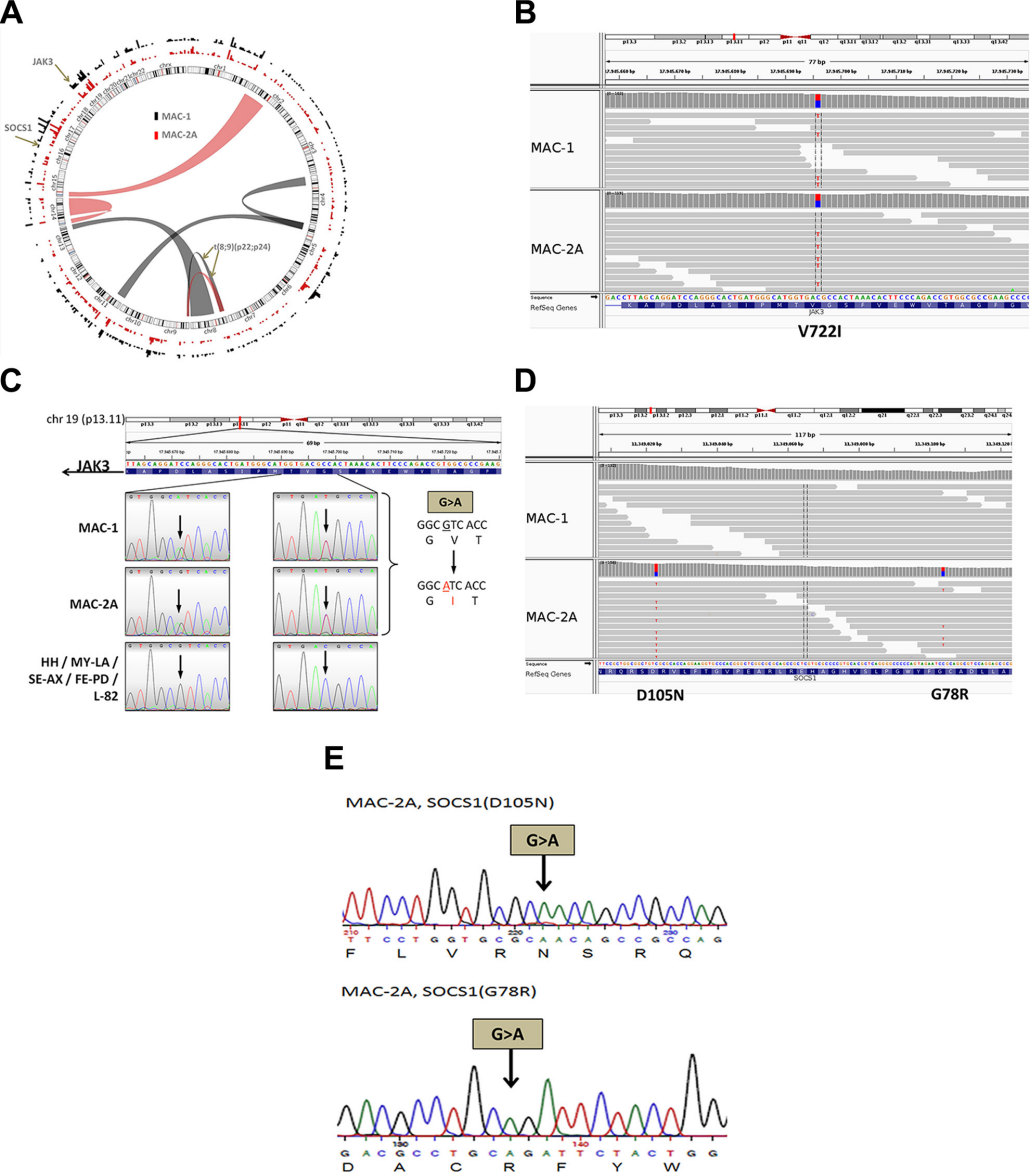
MAC cell lines carry JAK3 and SOCS1 mutations (**A**) Mutatome of MAC cell lines. Mutations ascribed serious consequences (frameshift, missense or chain terminating) are shown on the perimeter. Bar heights show mutation frequency along each chromosome. Chromosomal rearrangements are shown inside. Note t(8;9)(p22;p24) effecting fusion of PCM1 with JAK2 common to all MAC cell lines where it promotes STAT5 phosphorylation [20]. (**B**) Integrative Genomics Viewer (IGV) file shows heterozygous JAK3 (V722I) mutation in MAC-1/2A (http://www.broadinstitute.org/igv/MutationData). This mutation lies within the pseudokinase domain of JAK3 and confers constitutive activation promoting increased growth and survival in hematopoietic neoplasia and heterologous systems. (**C**) Re-sequencing of JAK3, comparing MAC cell lines with other ALCL/CTCL cell lines (FE-PD, HH, L-82, MY-LA, SE-AX). Sequencing panels show reverse (left) and forward (right) strands. Note the double (A/G) peak in MAC-1/2A in the left panel, as well as the purple double (C/T) peak in the right panel, indicating heterozygous mutation of JAK3. (**D**) IGV file shows SOCS1 mutations D105N and G78R. Note placement of mutations on opposite strands forming a compound heterozygote in repulsion. (**E**) Equivalent re-sequencing analysis for the SOCS1 mutations in MAC-2A. Representation shows that D105N and G78R mutations are compound heterozygotes.

Despite sharing identical TCR sequences [18] and primary cytogenetic alterations [20], preliminary experiments showed that MAC-1/2A displayed significant transcriptional diversity (Supplementary Figure 1A, 1B). To investigate the possible role of oncogenic mutations behind phenotypic evolution, whole exome sequencing was performed. This revealed 626 rare (allele frequencies < 0.01) nonsynonymous single nucleotide polymorphisms (SNP), notably frameshift, missense or chain terminating mutations, classed potentially harrmful by the Polyphen (http://genetics.bwh.harvard.edu/pph2/), Sift (http://sift.jcvi.org/) and Condel (http://omictools.com/consensus-deleteriousness-score-of-missense-snvs-tool) annotation tools. Alterations are summarized in Figure 1A. Whole exome sequencing revealed several distinguishing MAC-1 from MAC-2A reflecting their distinct disease-stage origins to serve as candidates driving or driven by disease progression, notably JAK3 (Figure 1B) verified by Sanger sequencing (Figure 1C); and SOCS1 (Figure 1D) again verified by Sanger sequencing (Figure 1E).

MAC-1 and MAC-2A carry heterozygous JAK3 mutations at chr19: 17,945,696 (C- > T) with allelic depths of 0.530 and 0.500, respectively, but absent from other ALCL/CTCL cell lines (FE-PD, HH, L-82, MY-LA, SE-AX). The identical mutation was also present in MAC-2B established alongside MAC-2A, from a separate skin lesion with an allelic depth of 0.524 (not shown). This gain-of-function mutation JAK3 (V722I) replaces valine with isoleucine at position 722 inside the pseudokinase domain leading to constitutive JAK3 activation, as described in megakaryoblastic, prolymphocytic and in T-cell acute lymphoblastic leukemias [5, 7–10].

While the JAK3 mutation was common to all three MAC cell lines, the SOCS1 gene carried two mutations in aggressive stage MAC-2A cells: at chr16: 11,349,104 (C- > T/allelic depth = 0.362), and 11,349,023 (C- > T/allelic depth = 0.569) (Figure 1D, Supplementary Table 1). Again the same lesion was present in MAC-2B where the corresponding allelic depths were 0.659 and 0.510 (not shown) while MAC-1 cells tested wild type. Re-sequencing confirmed that these mutations are compound heterozygotes, i.e. present in repulsion (Figure 1E). The predicted respective SOCS1 amino acid changes are G78R and D105N, i.e. exchanging the amino acids glycine 78 for arginine, and aspartic acid 105 for asparagine. Both mutations are novel and affect the SH2 (JAK binding) domain of SOCS1 [13], plausibly impairing interactions between SOCS1 and JAK kinases - a key question addressed experimentally below.

### Indolent stage plasticity traded against Th17 cytokines in aggressive stage cells

**Figure 2:**
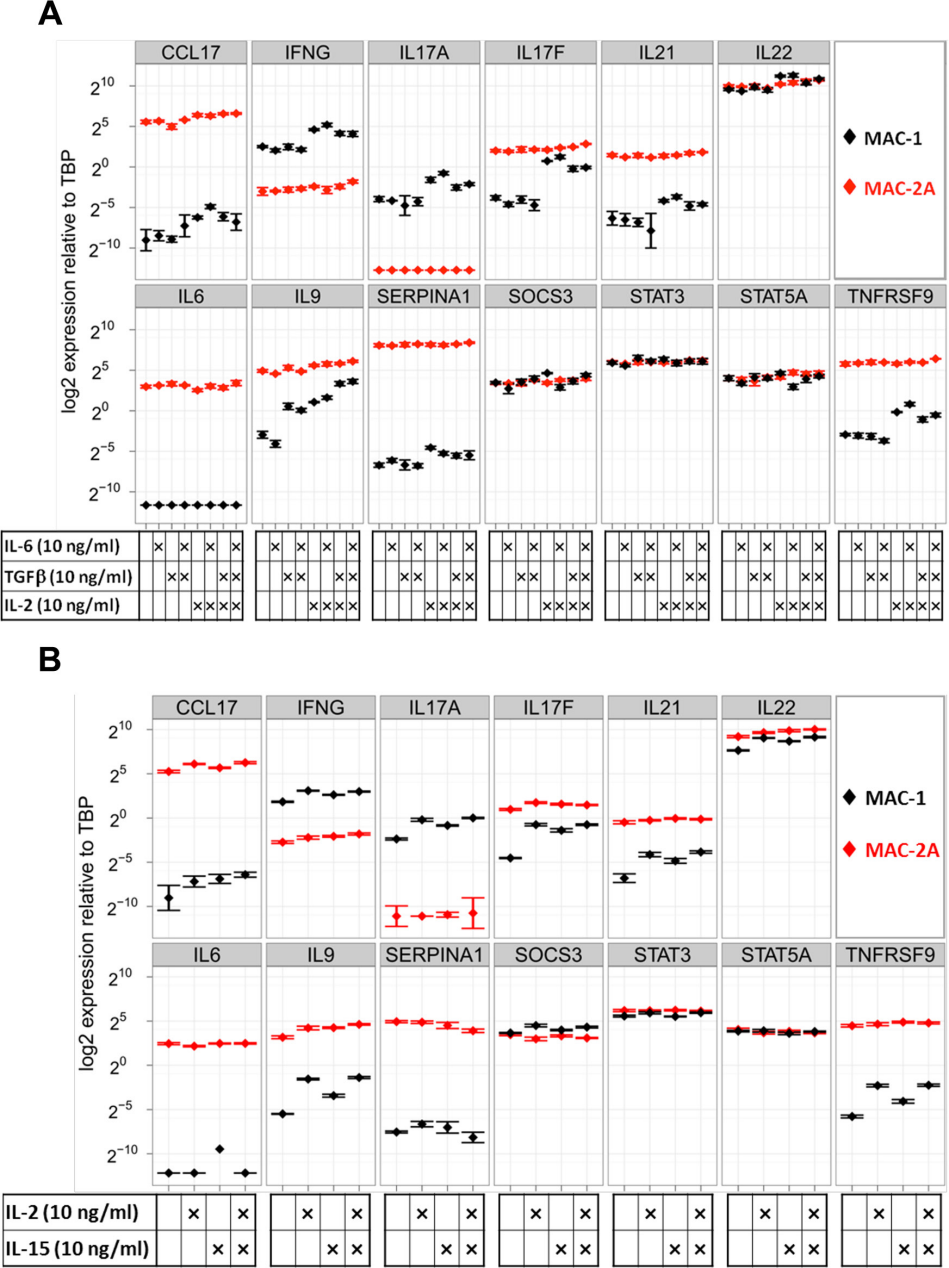
Common gamma-chain signaling through IL-2 and IL-15 drives Th17 cytokine expression in MAC-1 cells (**A**) Shows responses of MAC-1/2A to IL-2, IL-6, and TGFb. Note responsiveness limited to MAC-1 which showed transcriptional responses to IL-2 (notably, IL17A, IL17F, IL21, IL22, IL9, TNFRSF9), while IL-6 evoked no responses and TGFb responses were limited to the moderate induction of IL9 only in combination with IL-6. Thus MAC-1 cells showed wide-ranging plasticity limited to IL-2. (**B**) The transcriptional effects of single or combined IL-2 or IL-15 treatments show that IL-15 mimics the chief stimulatory effects of IL-2 on IFNG, IL17A, IL17F, IL21, IL22, IL9 and TNFRSF9, showing congruent cytokine signaling was employed. Cells were treated for 72 h and expression is shown relative to TBP in a logarithmic scale.

T-helper subsets, notably Th17 cells, display phenotypic plasticity [21–23]. Moreover, malignant CTCL cells may display phenotypic features of Tregs or IL-17 producing T helper (Th17) cells [2]. Since both JAK3 and SOCS1 genes are key players in STAT signaling in T-cell biology, we now addressed the phenotypic consequences of these mutations. Accordingly, we quantified the transcriptional responses in indolent and aggressive phase MAC cell lines to known T-cell differentiation factors, notably IL-2 reported to activate T-cells *in vivo* [21, 24]. Microarray expression profiling was used to investigate the global effects of exogenous IL-2, thus revealing several IL-2 responsive genes in MAC-1 matching those differentially upregulated in MAC-2A (Figure 2A, 2B, Supplementary Figure 1A).

We next tested the effects of IL-2 alone, or in combination with IL-6 and/or TGFβ. Unaided, IL-6 and TGFβ - classic agents for inducing Th17 differentiation, elicited minimal responses, limited to upregulation of IL9 by TGFβ (Fig 2A). In marked contrast, IL-2 treatment dramatically induced IFN-gamma, IL9, IL17A/F, IL22, TNFRSF9 and SERPINA1 in MAC-1 cells (Figure 2A). In the case of IL17F and IL9, IL-2 induced expression levels approaching those of MAC-2A cells, which, unlike MAC-1, remained stubbornly unresponsive to IL-2 (Figure 2A). Correspondingly unequal secretion of Th17 proteins between indolent and aggressive phase cells was confirmed by ELISA (Supplementary Figure 1B). Differentially expressed genes included IL9, IL17F, TNFRSF9 and SERPINA1, together with other T-cell regulators, including IL-6 and TGFβ. Thus, our data document induction of a wide spectrum of Th17 cytokines by treatment with IL-2, while the classical agents TGFβ and IL-6 proved largely or totally ineffectual.

Since IL-2 reportedly activates Treg signature genes [25], we also quantified expression of Treg markers FOXP3 and IL10, finding these unresponsive to IL-2 treatment (Supplementary Figure 2). Taken together, differentiation induced by IL-2 in MAC-1 was primarily directed towards the Th17 lineage and, while this observation contradicts the picture described in mice where IL-2 inhibits Th17 differentiation [26], it accords with the report that IL-2 promotes human Th17 cell expansion [24]. Intriguingly, the IL-2 inductive effect in MAC-1 recalls the tendency of inflammatory cutaneous neoplasms to undergo Th17 differentiation during disease progression [2].

IL-2 acts via the heterotrimeric IL-2 receptor, belonging to the common gamma-chain receptors and comprising the subunits IL2RA, IL2RB and IL2RG. Importantly, the common gamma-chain receptors are associated with JAK3, which upon stimulation initiate JAK-STAT signaling through STAT phosphorylation (STAT5A/B - Y694/Y699) [5]. Common gamma-chains, and also the IL-2 receptor beta-chain (IL-2RB), are shared between the IL-2 and IL-15 receptors which in turn display overlapping biological activities [27]. This prompted investigation of IL-15 treatment on the biology of MAC cells, showing it mimics the inductive effect of IL-2 on expression of IL-17F - and to a lesser extent of IL9 - in MAC-1. Moreover, combining IL-2 and IL-15 was not supra-additive (Figure 2B). Thus as foretold, IL-2 and IL-15 appear to operate in the same pathway in MAC-1 cells. The analysis of CTCL patient biopsies [28] further showed upregulation of genes that are subject to IL-2 mediated upregulation in MAC-1 cells and associated with aggressive phase (MAC-2A) expression profiles, revealing overall upregulation of IL15, JAK3, SOCS1 and TNFRSF9 or CCL17, IFN-gamma, IL17A/F, IL21, IL22, IL6 and IL9 in some patients (Supplementary Figure 3). In contrast to IL-2, IL-15 is produced in the skin and may therefore be more pathologically relevant [29]. Significantly, IL-15 but not IL-2 levels are elevated in MAC-2A cells (not shown) and in CTCL patients (Supplementary Figure 3), further supporting a clinical role for IL-15 signaling in CTCL progression.

To confirm transcriptional signaling by IL-2 or IL-15 conducted via their cognate receptors, we respectively quantified expression of inducible Th17 program genes in MAC-1 cells after IL-2 or IL-15 receptor blockade, respectively using IL-2RA or IL-15RA blocking antibodies. Antibodies against IL-2RA or IL-15RA both reduced expression of key genes, while the inductive effects of IL-2/IL-15 on, e.g. IFN-gamma or IL17F were partially abrogated after blockade of either receptor (Supplementary Figure 4). Collectively, the results of these experiments were consistent with a key signaling role for the common gamma-chain–JAK3 axis in governing phenotypic plasticity in MAC-1 cells.

### Gene expression in MAC cells requires intact JAK3/STAT5 signaling

**Figure 3:**
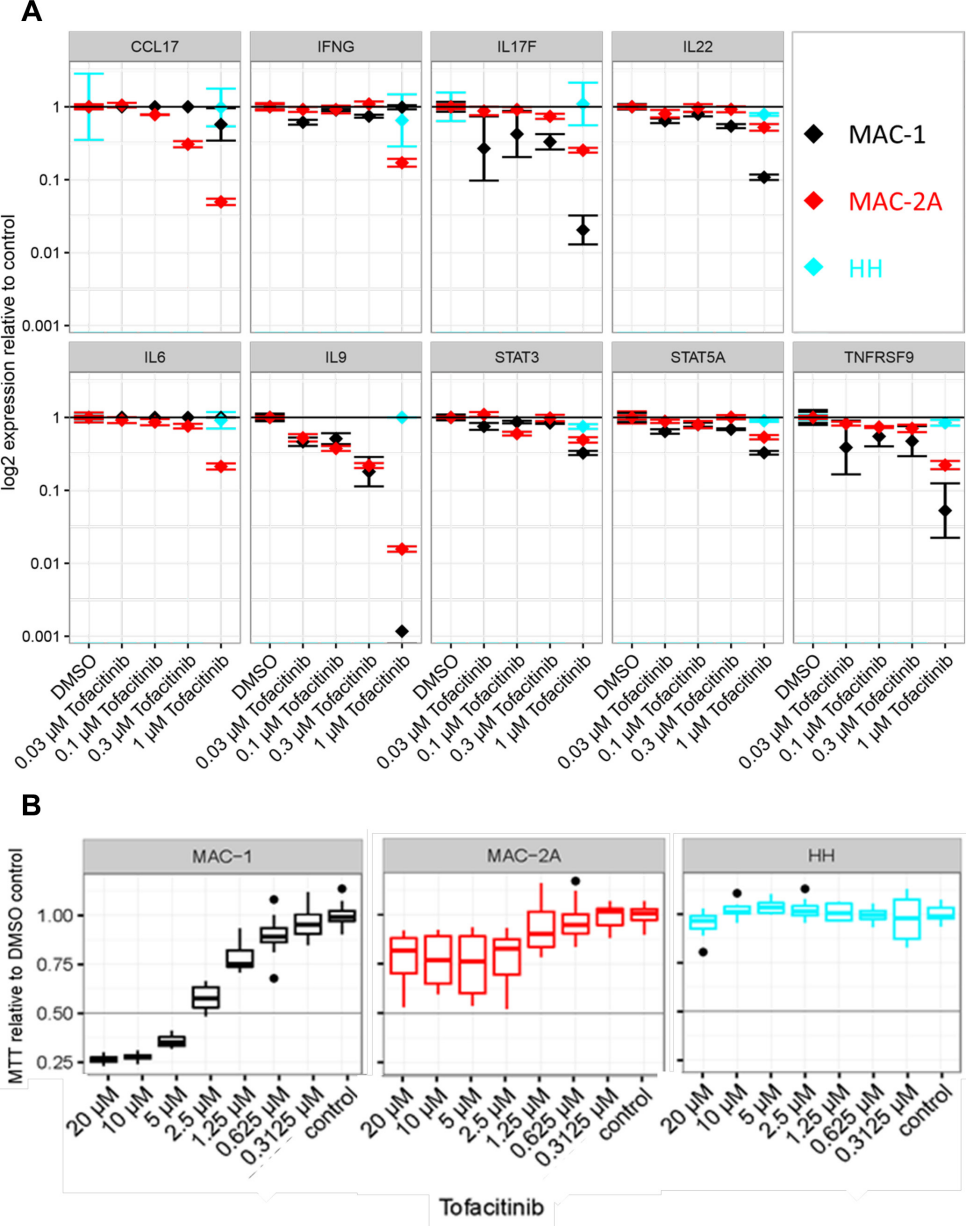

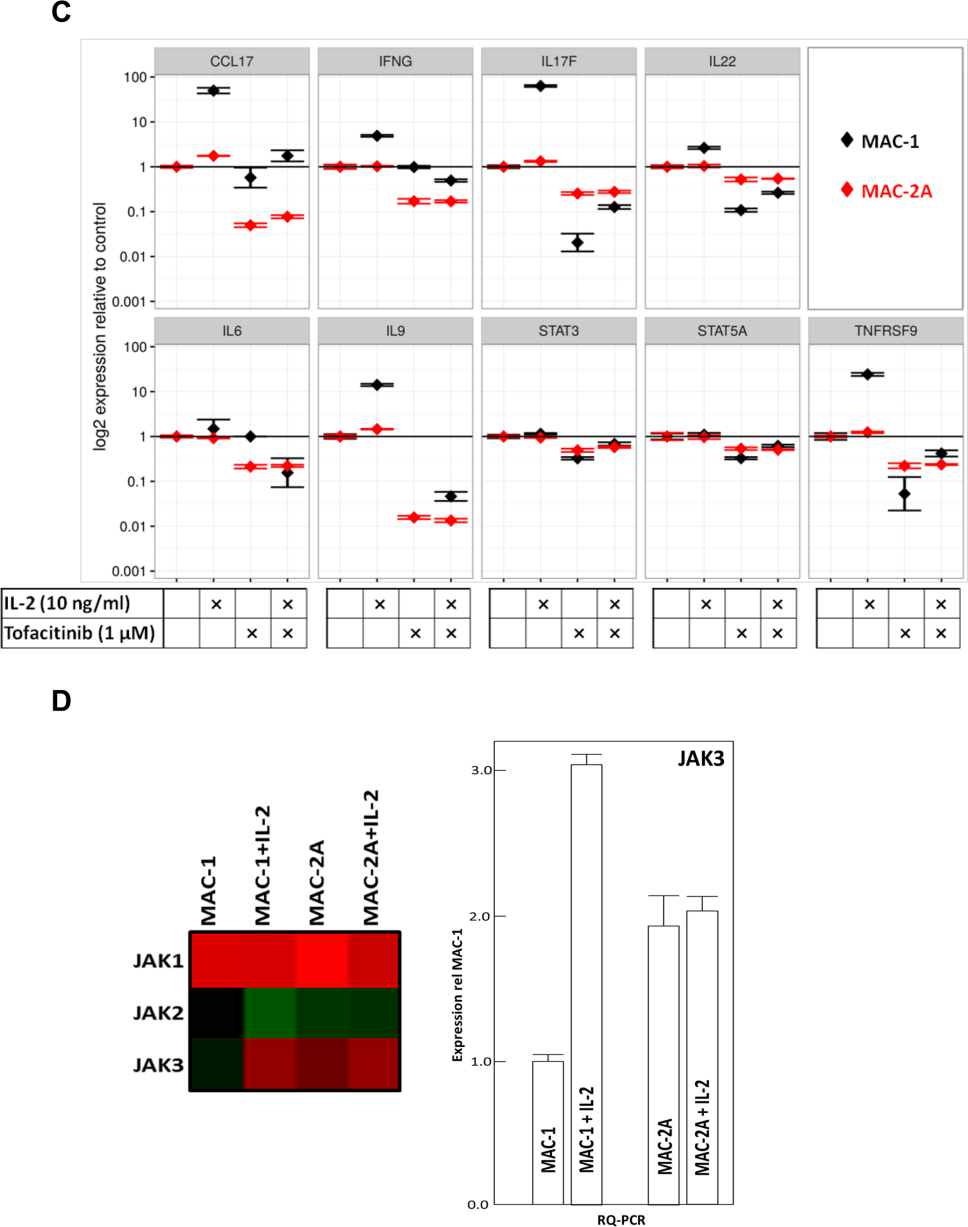

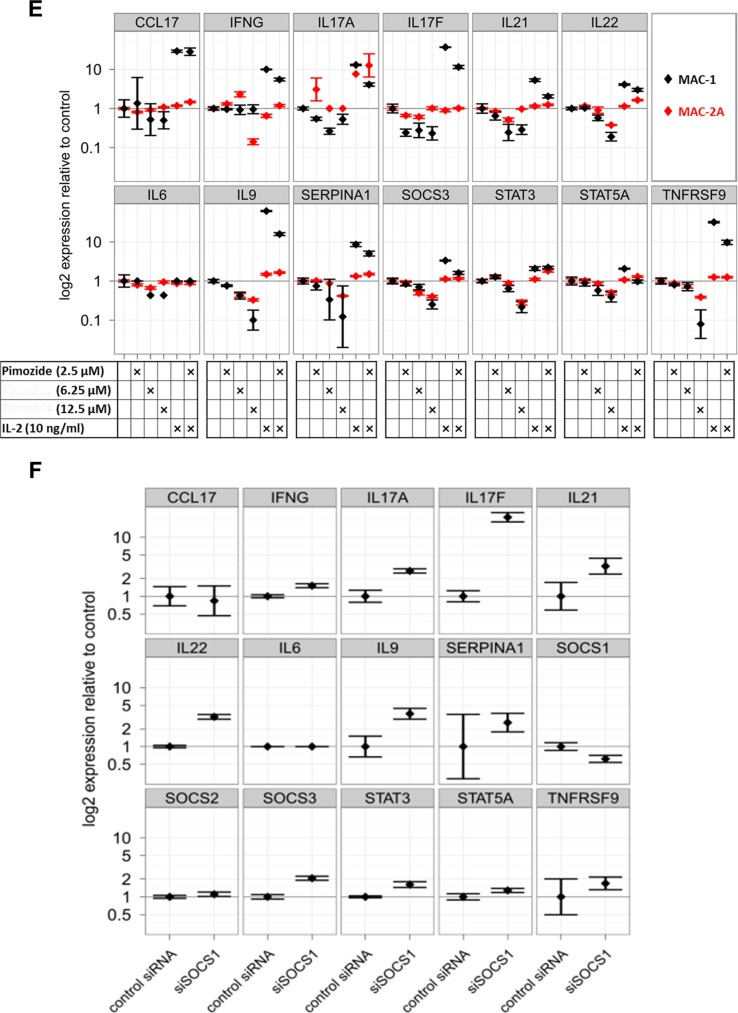
Th17 signature gene expression in MAC cells requires JAK3 signaling (**A**) Effect of dose dependent JAK3 inhibition on gene expression. Cells were treated with indicated levels of subtoxic doses of tofacitinib for 24 h and expression is shown relative to vehicle control in a logarithmic scale. Note unresponsiveness of HH cells to tofacitinib in contrast to MAC cells. (**B**) Effect of dose dependent JAK3 inhibition on cell viability. Cells were treated with indicated doses of tofacitinib for 72 h and cell viability was measured using MTT. Viability is shown relative to vehicle (DMSO) control. Note the dose response curve crossing the IC50 (0.5) line at 3 μM for MAC-1 and 20 μM for MAC-2A cells. (**C**) Antagonism of IL-2 driven gene induction by tofacitinib. Cells were treated with indicated levels of IL-2 and tofacitinib for 72 h. Note abrogation of IL-2 dependent transcriptional induction in MAC-1 by tofacitinib, while tofacitinib alone spares the same genes. (**D**) IL-2 stimulates JAK3 expression in MAC-1 cells. Left panel: Microarray heatmap shows depressed JAK3 expression in MAC-1 cells compensated by IL-2 treatment, while JAK-1/2/3 expression in MAC-2A cells remains unresponsive. Note IL-2 resistant constitutively high JAK1 expression in MAC-1/2A. Right panel: Quantification of JAK3 by RQ-PCR confirmed the microarray data showing circa threefold upregulation of in MAC-1 after IL-2 treatment even surpassing MAC-2A which again proved unresponsive. Cells were treated with 10 ng/ml IL-2 for 72 h. (**E**) Effect of STAT5 inhibition on gene expression and IL-2 dependent gene induction. Cells were treated with indicated levels of IL-2 and/or pimozide for 72 hours and relative expression is shown as above. Note abrogation of IL-2 dependent transcriptional induction in MAC-1 by pimozide. (**F**) Induction of gene expression in MAC-1 cells through siRNA mediated SOCS1 knockdown. Gene expression is shown relative to control siRNA as above. Note how SOCS1 knockdown mimics inductive effects of IL-2 on Th17 signature genes.

Given the central importance of JAK3 signaling in T-cell biology [30], we now investigated which key genes in CTCL cells require JAK3 activity. Hence, we treated MAC-1/2A and JAK3wt HH cells with the JAK3 inhibitor tofacitinib [31], which dose-dependently inhibited expression of several key genes including CCL17, IL17F, and IL9, and more moderately, IFN-gamma, IL22, IL6, and IL9. However, responses were uneven: preferentially targeting IL17F, IL22, IL9, TNFRSF9 in MAC-1, but IL6 and CCL17 in MAC2A (Figure 3A). These findings were consistent with a key signaling role for the constitutively activated JAK3 (V722I) allele; its presence in unmutated form in HH consistent with tofacitinib resistance (Figure 3A).

These conclusions were further supported by the proliferation-resistance of JAK3wt HH cells to tofacitinib (Figure 3B) which contrasted with the hypersensitivity of MAC-1 (IC50 = 3 μM) when compared to MAC-2A (IC50 = 20 μM) (Figure 3B).

Since the effects of tofacitinib and IL-2 are antagonistic and coupled via the common gamma-chain receptors, we investigated transcriptional differences after combined IL-2 and tofacitinib treatments. Significantly, the IL-2 driven induction of gene expression in MAC-1 was abrogated by tofacitinib (Figure 3C). Conversely, transcriptional downregulation with tofacitinib was blunted by IL-2, consistent with IL-2 regulation of gene expression in MAC-1 via JAK3. However, the inhibitory effects of tofacitinib were only marginally rescued by IL-2 in MAC-2A cells when compared to MAC-1 where expression of most Th17 cytokines, including IL17F, IL22, IL9, STAT3, STAT5A and TNFRSF9 was significantly restored (Figure 3C). This discrepancy was taken to imply that unlike MAC-1, JAK3 expression in MAC-2A cells was independent of IL-2 stimulation. This notion was supported by higher native JAK3 mRNA expression in MAC-2A than MAC-1, a discrepancy more than compensated by IL-2 treatments which left JAK3 levels in MAC-2A cells scarcely perturbed (Figure 3D left and right panels). In contrast, identical IL-2 treatments failed to induce JAK1 or JAK2 in MAC-1 and MAC-2A cells alike (Figure 3D left panel).

Because the IL-2 - common γ-chain receptor - JAK3 cascade also promotes STAT5 phosphorylation [5], we next investigated the effects of STAT5 inhibition using the selective STAT5 inhibitor pimozide. Significantly, pimozide treatment lowered the expression of IL17A, IL17F, IL21, IL22, IL9, SOCS3, STAT3, STAT5 and TNFRSF9 in MAC-1, but also in MAC-2A cells albeit less dramatically (Figure 3E), showing that expression of signature genes in MAC cells was STAT5 dependent. Since pimozide and IL-2 act antagonistically, we also tested the effects of combining both reagents. Interestingly, the inductive effect of IL-2 on IFNG, IL17F, IL22, IL9, SOCS3, STAT5A and TNFRSF9 was muted by pimozide in MAC-1 cells (Figure 3F), confirming that IL-2 dependent gene induction in MAC-1 involves STAT5.

### SOCS1 mutations in MAC-2A weaken critical JAK3-SOCS1 interaction

SOCS1 binds to JAK via the SH2 domain, forming a complex which inhibits IL-2 induced STAT5 phosphorylation [15, 32]. Since MAC-2A carries SOCS1 mutations within the SH2 domain and expression of signature cytokines is high but resistant to further induction through IL-2, we hypothesized that defective SOCS1 inhibition is mutationally incapacitated in these cells. To test a prediction of this model, namely that inhibition of SOCS1 in MAC-1 cells enhances STAT5 dependent gene expression, we performed siRNA mediated SOCS1 knockdown. This showed an IL-2-reminiscent inductive effect on gene expression through SOCS1 knockdown in MAC-1 cells (Figure 3E), suggesting that SOCS1 mediates inhibition of IL-2 – JAK3 – STAT5 signaling.

**Figure 4:**
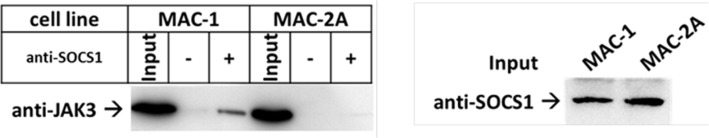
JAK3-SOCS1 interaction disrupted by SOCS1 mutations (**A**) Western blot shows co-IP of JAK3 and SOCS1. Cell lysates of MAC-1 (SOCS1wt) and MAC-2A (SOCS1 mu/mu) were incubated with anti-SOCS1 antibodies and tested for the presence of JAK3. Note the stronger interaction between JAK3 and SOCS in MAC-1 when compared to MAC-2A. Western Blots show co-IP and another SOCS1 blot replacing the precipitation control, due to overlapping signals of SOCS1 and the IgG light chain. Western blots fairly represent two independent biological replicates.

**Figure 5:**
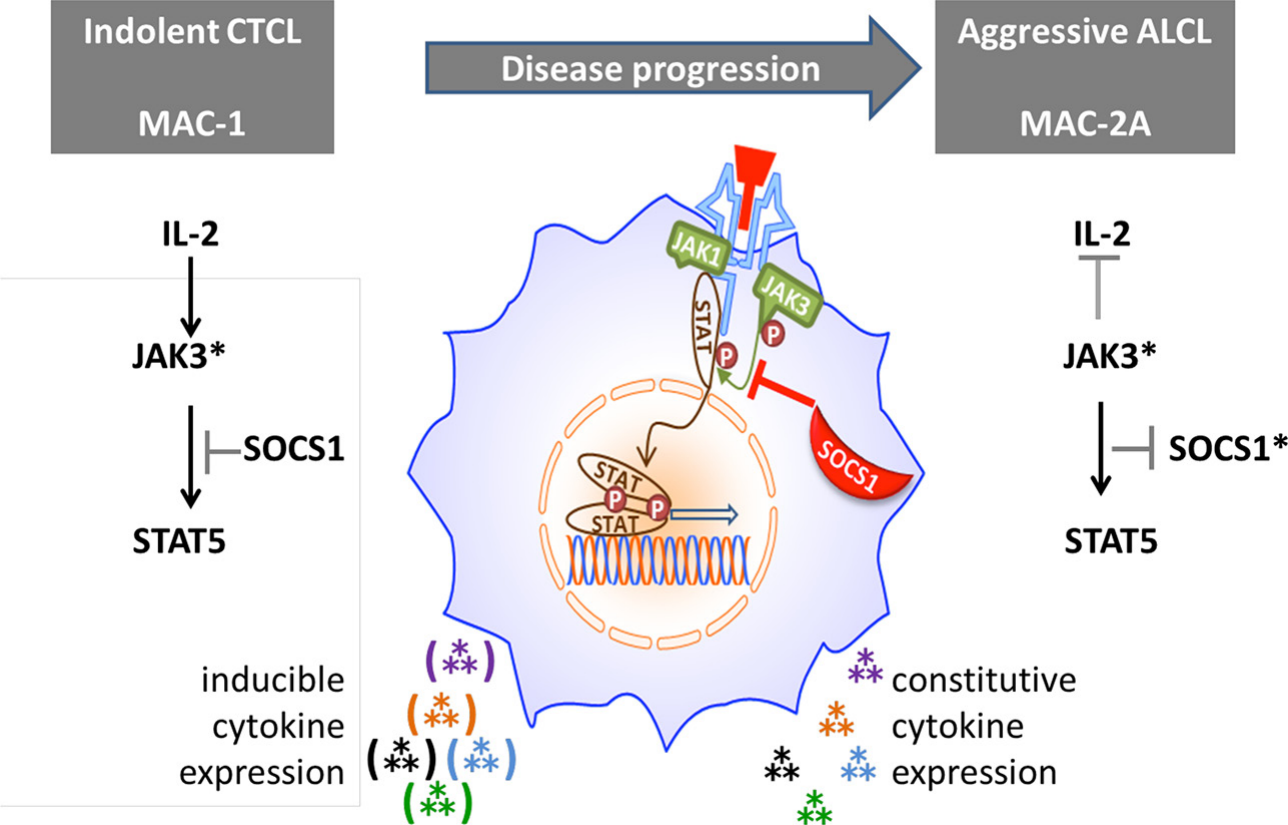
Proposed role of SOCS1 in modulating cytokine signaling via the JAK/STAT axis Hence, regulation of constitutive JAK3 mutational (*) activation of Th17 cytokine expression by wt SOCS1 (left figure) is thwarted by SOCS1 inactivating mutations formed during disease progression (right). However, IL-2 signaling can circumvent negative regulation of JAK3* by SOCS1 in MAC-1 cells by boosting JAK3.

To further test this model, we compared the JAK3 binding efficiencies of SOCS1 G78R/D105N (in MAC-2A) and wt SOCS1 (in MAC-1) to JAK3 in co-immunoprecipitation (IP) experiments. This exercise confirmed interaction between wt SOCS1 and JAK3 in MAC-1 cells, as shown by the presence of JAK3 in SOCS1 precipitated fractions (Figure 4). This interaction product was absent in MAC-2A cells, indicating that SOCS1 G78R/D105N mutations abolish SOCS1-JAK3 binding required for suppression. Hence, our data identify two key amino acid substitutions (G78R, D105N) inside the SH2 JAK binding domain, each essential to the binding integrity of SOCS1. Thus, constitutive JAK3 activation via V722I mutation can be compensated in MAC-1 cells through wt SOCS1, while the acquisition of SOCS1 G78R/D105N mutations abolish the SOCS1 mediated regulation of the common gamma-chain – JAK3 – STAT5 axis in MAC-2A cells (Figure 5). Collectively, our findings show that constitutive JAK3 activation via V722I mutation in concert with SOCS1 G78R/D105N mutations and the previously reported t(8;9) PCM1-JAK2 fusion which operates via JAK2 [20] represent three JAK-STAT activation modes, variously impacting malignant and inflammatory phenotypes in CTCL cells both alone and in concert.

## DISCUSSION

Here we characterize a dual lymphoma progression/candidate Th17 cell line model. Our model is two-stage - “before-and after”, comprising cell lines respectively established at indolent and aggressive disease stages providing controlled inducible cytokine expression which vividly recalls the inflammatory cytokine profiles seen at advanced CTCL. Using this model we reveal a two-step mutational process reflecting disease progression, whereby an activating JAK3 mutation known to drive lymphoma formation is joined in progressed disease by novel inactivating mutations of SOCS1, thereby weakening the latter's inhibitory potency. We further show that expression of a core inflammatory element - one reportedly associated with progressed CTCL, namely IL17F, can be induced by SOCS1 knockdown performed in indolent disease (MAC-1) CTCL cells bearing wild type SOCS1. Co-IP experiments showed that the SOCS1 mutations in question impaired binding to JAK3 upon which their inhibitory interactions are known to be contingent, thus providing a cogent explanation for exuberant JAK3 downstream cytokine signaling in MAC-2A cells bearing both SOCS1 mutations. As a corollary, the Th17-like cytokine profile seen in aggressive disease MAC-2A cells was partially abolished by pharmacological inhibition of JAK3 or STAT5 implicating this signaling pathway in inflammatory disease progression. It should be noted, however, that doubts have been raised over the reported selectivity of pimozide, implying the need to verify conclusions based on the use of this inhibitor alone. Moreover, the wide Th17 profile seen in aggressive stage cells was readily inducible in plastic, indolent stage MAC-1 cells by IL-2, a cytokine reportedly inimical to Th17 differentiation - while “classical” IL-6/TGFβ treatments proved ineffectual. And although MAC-2A cells secrete copious amounts of IL-6, antibody blockade of IL6R (tocilizumab) effected only moderate (circa twofold) inhibition of IL17F expression (Ehrentraut et al., in preparation). Thus collectively, MAC-1/2A cells comprise a dynamic *in vitro* system for modelling lymphomatous progression and inflammatory cytokine differentiation driven by JAK/STAT pathway mutations which may be recapitulated using IL-2 and which highlights this cytokine in driving IL-17F expression, at least in the cell contexts described herein.

Isogenic systems like the T-cell neoplasia progression model described here - the first reported hitherto - help distinguish indictable mutations from neutral polymorphisms. Key phenotypes studied here were the plasticity of indolent phase cells in response to IL-2, traded against the exuberant cytokine expression of aggressive phase cells, matching that described clinically [3, 33, 34]. Both, IL-2 and IL-15 reportedly promote growth and viability of CTCL and Sézary syndrome derived cell lines, while co-expression of IL-15 and IL-17F characterizes mycoses fungoides [35]. In contrast to IL-2, IL-15 is secreted in normal skin as a chemoattractant for activated T-lymphocytes thereby recruiting malignant CTCL cells and sustaining their cutaneous growth and may well be the pathologically relevant cytokine here [35].

Both JAK3 and SOCS1 mutations involve C > T transitions occurring at CpG sites. C > T enrichment of mutational signatures, for which our sequencing data provide some support, has been attributed to spontaneous deamination of 5-methylcytosines or DNA mismatch repair [36]. The primary gene alteration here - activation of JAK3 via a recurrent mutation JAK3 (V722I) - was previously reported in a variety of hematopoietic neoplasms as a driver mutation enabling factor-independent growth [10]. The second alteration, acquired at aggressive disease - compound heterozygous mutation of SOCS1 (G78R/D105N) – is new. It is worth asking to what extent may *in vitro* artifact be excluded. Accordingly, the JAK3 (V722I) mutation occurred in three karyotypically distinguishable cell lines studied at early passage: MAC-1/2A as described here plus MAC-2B established along with MAC-2A from a separate skin lesion (data not shown). These observations imply earlier formation in the putative neoplastic stem cell compartment, while rendering implausible an artifactual origin *in vitro*. Moreover, the identical mutation has been described in a variety of hematopoietic neoplasms, including T-cell lymphoma [7]. The SOCS1 mutations are novel, however, but being present in identical form in both MAC-2A as described above and MAC-.2B (not shown), an *in vivo* origin is also shown, again in an evolving stem cell. In short, given their multiple occurrence an *in vivo* origin for both sets of mutations is warranted, plausibly in a neoplastic stem cell.

Assembling these findings into a unified picture (Figure 5), we propose that IL-2 signaling via the gamma chain receptor sufficiently boosts expression of mutationally activated JAK3 to overcome inhibition by SOCS1wt in MAC-1 cells. But in MAC-2A cells, mutationally disabled SOCS1 lets JAK3 activation proceed unimpaired, independently of IL-2 stimulation. Unimpeded JAK3/STAT5 signaling, following SOCS1 mutation (MAC-2A) or knockdown (MAC-1), is accompanied by vivid cytokine expression, notably IL17F. Of course, such a model necessarily ignores additional impactful changes, some acquired during disease progression, e.g. that affecting FBXW7 in aggressive stage cells which impacts NOTCH signaling. The effects of these additional mutations will be addressed separately.

Although IL2RG – JAK3 – STAT5 signaling may involve JAK1, the last is usually activated via the IL-2 beta chain receptor [5, 9]. Moreover, mutations affecting JAK1 or IL2RG were absent from MAC-1/2A, as were nearby genomic copy number or cytogenetic alterations, collectively suggesting unaltered signal transduction through JAK1, reinforcing the claim of JAK3 as the principal conductor of IL-2 signaling. Confirming this notion, JAK3 in MAC-1 cells was boosted to MAC-2A levels or above by IL-2 treatments which spared JAK1/3. Both MAC-1 and MAC-2A cell lines were conspicuously sensitive to JAK3 inhibition which concurrently attenuated proliferation and Th17 cytokine expression in MAC-1 cells together with IL-2 responses. STAT5 inhibitory treatments produced similar effects, further indicting JAK3-STAT5 signaling as the main highway involved in inflammatory cytokine production in these cells.

Eschewal of T-regulatory in favor of Th17 differentiation in the CTCL system described here may be partly attributable to co-induction of IL-21 which is known to promote IL-17 expression at the expense of FOXP3 [37]. Collectively, these findings show coupling of both plasticity and inflammatory differentiation to JAK-STAT signaling and indict SOCS1-JAK3 binding as a key regulatory chokepoint.

SOCS1 inactivating mutations or deletions have been previously shown to abolish control of phospho-JAK2 or phospho-STAT5 signaling in primary mediastinal B-cell and Hodgkin lymphomas [16, 17], recalling the picture described here for JAK3 and SOCS1. Therefore, loss of SOCS1 activity and its attendant negative regulation of activated JAK3 represent another mode of JAK/STAT activation. Recently it was reported that in ALK-negative ALCL, SOCS1 expression is regulated by miR-155 [38]. However, a role in phenotypic divergence in MAC-1/2A is unlikely, since although miR-155 expression in MAC-1 and MAC-2A is high, SOCS1 protein levels were similar in both cell lines, again highlighting the significance of mutational SOCS1 inactivation described here. Consistent with these findings, overexpression of SOCS1 reportedly inhibits IL-2 induced STAT5 phosphorylation via repressive JAK3 or JAK1 binding [32].

Keeping step with IL17F and IFN-gamma, TNFRSF9 (also known as 1–4BB or CD137) was conspicuously upregulated by IL-2 treatment. Induction of TNFRSF9 by ionomycin or phorbol 12-myristate 13-acetate (PMA) has already been described [39]. TNFRSF9 is a costimulatory factor which promotes survival and inflammatory secretion in T-cells though few details are known [40]. Hence, our findings uncover a mechanism of potential therapeutic interest, namely, induction of immunogens or other actionable targets in cancer cells by treatments with nontoxic agents.

While this study has focused on STAT5, STAT3 is also implicated in IL-2 signaling via JAK3 in driving IL-17 expression in CTCL [33]. We recently reported that in MAC-cells STAT3 phosphorylation and contingent STAT5 upregulation require PCM1-JAK2 formation by a recurrent chromosome rearrangement t(8;9)(p22;p24) which yields constitutively activated JAK2 protein [20]. Both pharmacological inhibition of JAK2 using TG-101348 and PCM1-JAK2 knockdown decreased pSTAT3 protein and STAT5 expression, showing that STAT3 and STAT5 in MAC cells act in concert. Clearly, additional studies are required to disentangle crosstalk across JAK/STAT signaling in CTCL, notably the respective contributions of STAT3 and STAT5 to oncogenesis and inflammatory gene expression attending disease progression.

Our picture of T-cell plasticity derives from nonmalignant cells, primarily in mouse, as do the key features of main T-helper classes (such as, Th9, Th17, Th22, etc). However, the applicability of these defining characteristics to humans is uncertain. Moreover, the original clear picture of signature cytokines distinguishing four key Th-subsets, Th1, Th2, Th17 and Tregs, has become hazier and multidimensional with the recognition of additional overlapping categories and transgressive plasticity. The most notable discrepancy reported here is the Th17 promotional role of IL-2 and ineffectuality of IL-6 which contradicts that already described in mice. Whether such discrepancies are restricted to CTCL or occur more generally among human Th cells requires additional investigation.

This study extends the emerging paradigm of JAK3 activating mutations [5, 7, 41] to CTCL and further implicates SOCS1 in the control of lymphomatous signaling, thus widening the number of potentially actionable targets in affected neoplasms. Accordingly, we believe our findings justify targeted screening for dual JAK3/SOCS1 mutations in CTCL at both early and progressed disease stages. These data both proffer a molecular mechanism for the inflammatory Th17 signatures known to accompany CTCL disease progression, and endorse MAC-1/2A cells as a preclinical model. MAC-1/2A cells also provide a system for investigating signaling pathways involved in IL-17F expression driven by IL-2/15 - a novel signaling pathway. Moreover, given their widespread involvement in inflammatory disease and cancer, the MAC-1/2A system provides a candidate human Th17 model for evaluation, notably to determine the pathophysiological relevance of signaling networks therein, such as that described here. Finally, the exquisite responsiveness of MAC-1 cells (and other CTCL cell lines currently under investigation) to IL-2 and diverse sub-toxic agents in expressing potentially actionable targets, such as TNFRSF9 [42], implies that CTCL cell lines are worthy of further evaluation to determine the potential usefulness of such an approach. All in all, these remarkable cells raise more issues than they settle, including the axiomatic sacrifice of differentiation during cancer progression.

## MATERIALS AND METHODS

### Cells and patients

Authenticated leukemia/lymphoma cell lines established from patients with ALK-positive ALCL (L-82); ALK-negative ALCL (FE-PD), and CTCL (HH, MY-LA, SE-AX – the latter two kindly donated by Dr Keld Kaltoft, University of Copenhagen) - are detailed in Drexler [19] and drawn from the DSMZ public and research repositories. MAC-1/2A/B cell lines were established by an author (MEK). Clinical particulars of the MAC-donor patient are given by Davis et al. [18] and summarized in [20]. Briefly, MAC-1 cells were established in 1985 at the indolent stage of a cutaneous T-cell lymphoma, followed in 1987 by MAC-2A after progression to fatal (CD30+) ALCL. Morbid and healthy donor gene expression data were from ref. 28 accessed as GEO data set GSE59307. All cell lines were authenticated by DNA STR profiling augmented by cytogenetic analysis to verify isogenic MAC-1/2A which are karyotypically distinct [20].

### Cell culture

Cells were cultured as recommended (http://www.dsmz.de). Treatment of cell lines with effector molecules/cytokines/chemicals was performed with indicated factors for various incubation times. For transfer of nucleic acids 80 pmol siRNA construct directed against SOCS1 was tranfected into 1 × 10^6^ cells using the EPI-2500 impulse generator (Fischer, Heidelberg/Germany) at 350 V for 10 ms [43].

### Reverse transcriptase (RT-) and quantitative real-time (RQ-) PCR

Total RNA from cell lines or cells treated with indicated factors for the indicated time was extracted using the RNEasy Kit (Qiagen, Hilden/Germany). cDNA was subsequently synthesized from 3 μg RNA by random priming using Superscript II (Invitrogen). RQ-PCR was performed by the 7500 Fast Real-time PCR System (Applied Biosystems, Darmstadt/Germany) using SsoFast EvaGreen Real Mastermix (BIORAD, Hercules, CA/USA) and custom designed oligonucleotides (MWG Eurofins (Martinsried/Germany - sequences available upon request). For normalization of expression levels we used TATA box binding protein (TBP). Quantitative analyses were performed in triplicate, repeated on two or more occasions. The analysis of relative quantitative expression was performed using the Applied Biosystems software, followed by statistical analysis and data visualization using the R-based ggplot2 package [44].

### Global gene expression arrays

To parse gene expression, we generated a cDNA expression array using the Affymetrix HGU133plus2 Chip (Santa Clara/CA). For statistical analyses of primary expression data (CEL-files) and subsequent generation of heat maps we used the R-based Bioconductor Linear Models for Microarray Data (LIMMA) package [45]. For the latest release of gene annotation data we used the Bioconductor homepage (http://www.bioconductor.org). Minimum Information about a Microarray Experiment (MIAME) is available upon request for all microarray experiments. Gene ontology and pathway analyses were performed using the Broad Institute Gene Set Enrichment Analysis (http://software.broadinstitute.org/gsea/index.jsp) and DAVID [46] platforms, respectively.

### Protein analysis and co-immunoprecipitation (co-IP)

Western blot analysis was performed by the semi-dry method. Proteins obtained from cell lysates were transferred onto nitrocellulose membranes (Bio-Rad, Munich/Germany) and blocked with 5% bovine serum albumin (BSA) or milk powder dissolved in phosphate-buffered-saline buffer (PBS). GAPDH served as loading control [47].

For co-IP experiments 1.5 × 10^8^ cells were lysed in (25 mM Tris/HCL pH 7.5, 150 mM KCL, 2 mM EDTA, 0.5 mM DTT, 0.5% Igepal CA-630 - Sigma Munich/Germany) containing protease inhibitors (Sigma). Lysates were precipitated using an anti-SOCS1 antibody (Sigma, AV42147) bound to protein-G-sepharose beads (Sigma). Eluted fractions were analyzed by Western Blotting using anti-JAK3 antibody (Santa Cruz, sc-6932, Heidelberg/Germany. Secondary antibodies were linked to peroxidase for detection by Western-Lightning-ECL (Perkin Elmer, Waltham, MA, USA). Image documentation employed the digital ChemoStar Imager system (INTAS, Göttingen, Germany).

Cytokine secretion was measured by enzyme-linked immunosorbent assay (ELISA). For analysis of IFN-gamma, IL-1A, IL-2, IL-4, IL-6, IL-12, IL-13, IL-17A, TARC, TGFβ1, TNFα, a customer designed Mix-N-Match ELISArray Kit (Qiagen) was used, analysis of IL-17F and IL-22 was performed with Quantikine ELISA Kits (R&D Systems, Wiesbaden-Nordenstadt/Germany) following the manufacturer's protocol. Briefly, 50 μl of each sample, control or standard were added to 96-well plates pre-coated with the relevant capture antibodies. After 2 h incubation, plates were washed thrice and 100 μl conjugated detection antibody added to each well, followed by 1 h incubation. Plates were washed and avidin-HRP added and incubated for 30 min, followed by four washes. Development solution was added and plates incubated for 15 min in the dark. Finally, stop solution was added and optical density measured by ELISA reader (Multiscan EX, ThermoFischer).

### DNA sequencing

Whole exome sequencing was performed by Oxford Gene Technologies (Oxford/UK), using the Illumina HiSeq platform. Enrichment and library preparation samples were prepared according to Agilent's SureSelect Protocol Version 1.2. DNA concentration of each library was determined using Agilent's QPCR NGS Library Quantification Kit (G4880A) and samples were adjusted to a final concentration of 10 nM. Mapping and alignment was performed using 89.9% of the on-target regions covered to a depth of at least 20 ×. A total 16.59 Gbp of sequence data were read and aligned at high quality and 268,493 variations from the reference genome were identified. Reads were mapped according to build hg19/b37 using the Burrows-Wheeler Aligner package, version 0.6.2. Local realignment was carried out with the Genome Analysis Tool Kit (Broad Institute GATK version 1.6). Base quality (Phred scale) scores were recalibrated using GATK's covariance recalibration. SNP and indel variants were called using the GATK Unified Genotyper for each sample. Genotyping includes dbSNP release 135 to improve quality of calls. SNP novelty was determined against dbSNP release 132.

For Sanger sequencing, genomic DNA was extracted using the Gentra Puregene DNA-Isolation Kit (Qiagen). PCR primers were obtained from MWG Eurofins (Ebersberg/Germany). Genomic PCR was performed using the C1000 Thermo Cycler (Biorad, Munich/Germany). PCR products were separated with 2% agarose gel and purified with the QIAquick Gel Extraction Kit (Qiagen). Purified PCR products were directly sequenced by MWG. PCR products for SOCS1 were cloned into pGEMT vector (Promega, Mannheim/Germany) for sequencing (MWG).

For a conspectus of gene rearrangements the Circos program was used [48]. Sequencing reads alignment and specific mutation sites for JAK3 and SOCS1 were plotted via Integrative Genomics Viewer (IGV) [49] and cytogenetic data incorporated [50].

## SUPPLEMENTARY MATERIAL FIGURES AND TABLE


