# Supplementary material for: Th17 cytokine differentiation and loss of plasticity after SOCS1 inactivation in a cutaneous T-cell lymphoma
Source: Oncotarget. 2016 Apr 28;7(23):34201–16. doi: 10.18632/oncotarget.9077 (PMC5085149; doi:10.18632/oncotarget.9077)
Supplement: Supplementary file 1 [file oncotarget-07-34201-s001.pdf]

Th17 cytokine differentiation and loss of plasticity after SOCS1 inactivation in a cutaneous T-cell lymphoma

Supplementary Materials

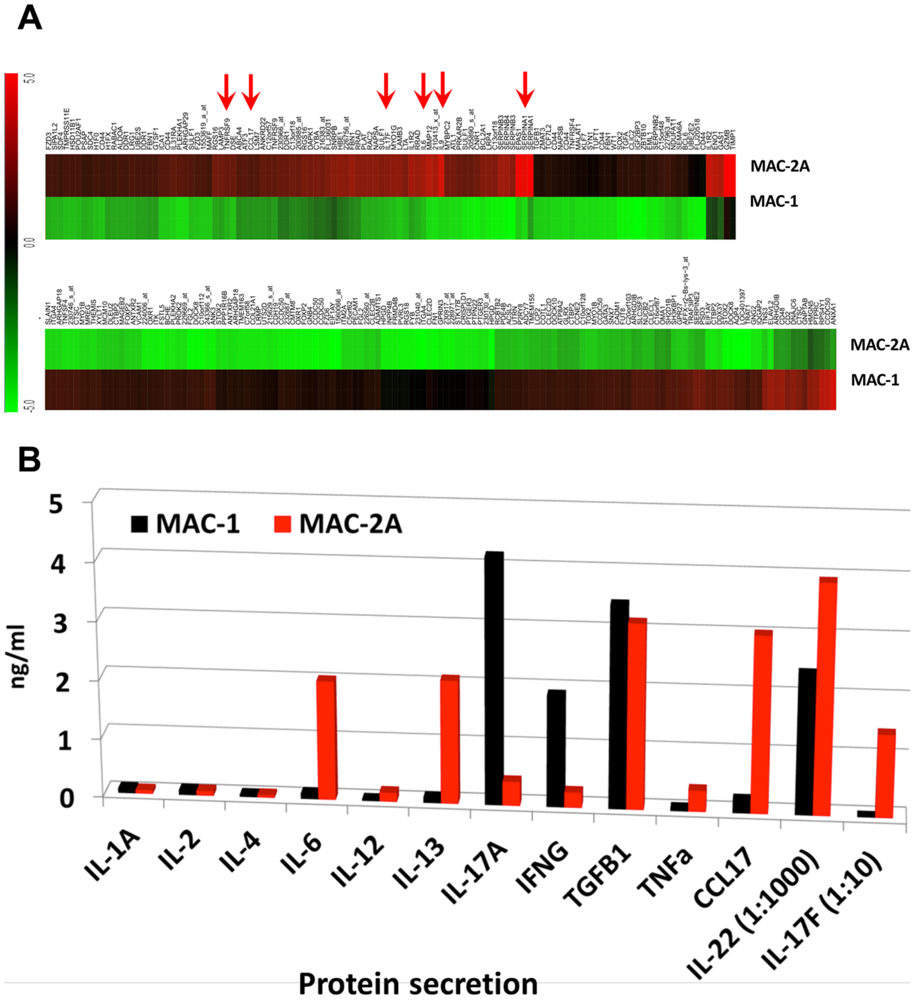

Supplementary Figure S1: Global differences in gene expression between MAC-1 and MAC-2A cell lines. (**A**) Heatmap showing differential gene expression comparing MAC-1 to MAC-2A cells. Arrows indicate genes conspicuously upregulated in MAC-2A. (**B**) ELISA shows differential expression in MAC-1 vs MAC-2A of secreted Th17 cytokines - notably IL-6, IL-17F, IL-22, together with pro-inflammatory proteins, such as CCL17. Although IL-17A secretion by MAC-1 cells is higher, amounts are dwarfed by IL-17F in MAC-2A.

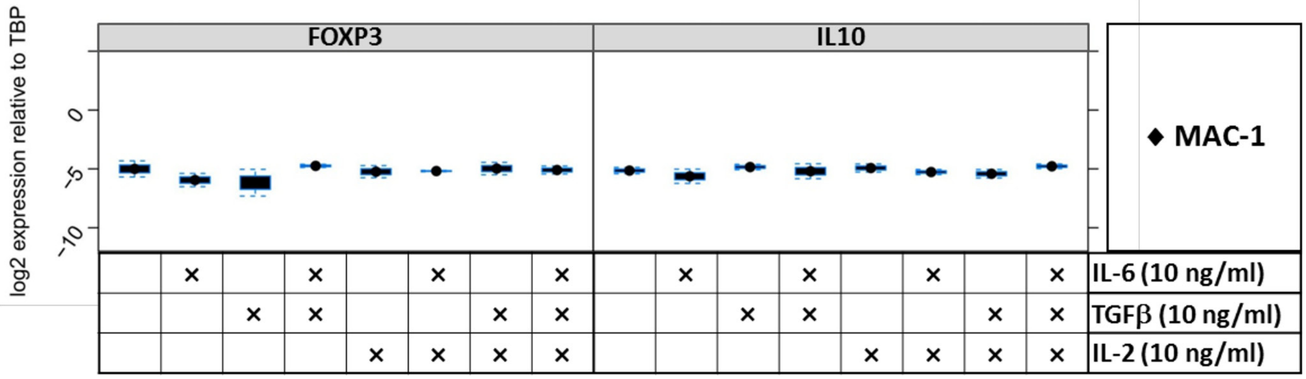

Supplementary Figure S2: Eschewal of treg characteristics in MAC-1. Shows ineffectiveness of IL-2, IL-6 or TGF-beta for inducing expression of key Treg signature genes FOXP3 and IL-10 in MAC-1 cells. Cells were treated at the doses indicated for 72 h. Expression after treatment is given relative to TBP.

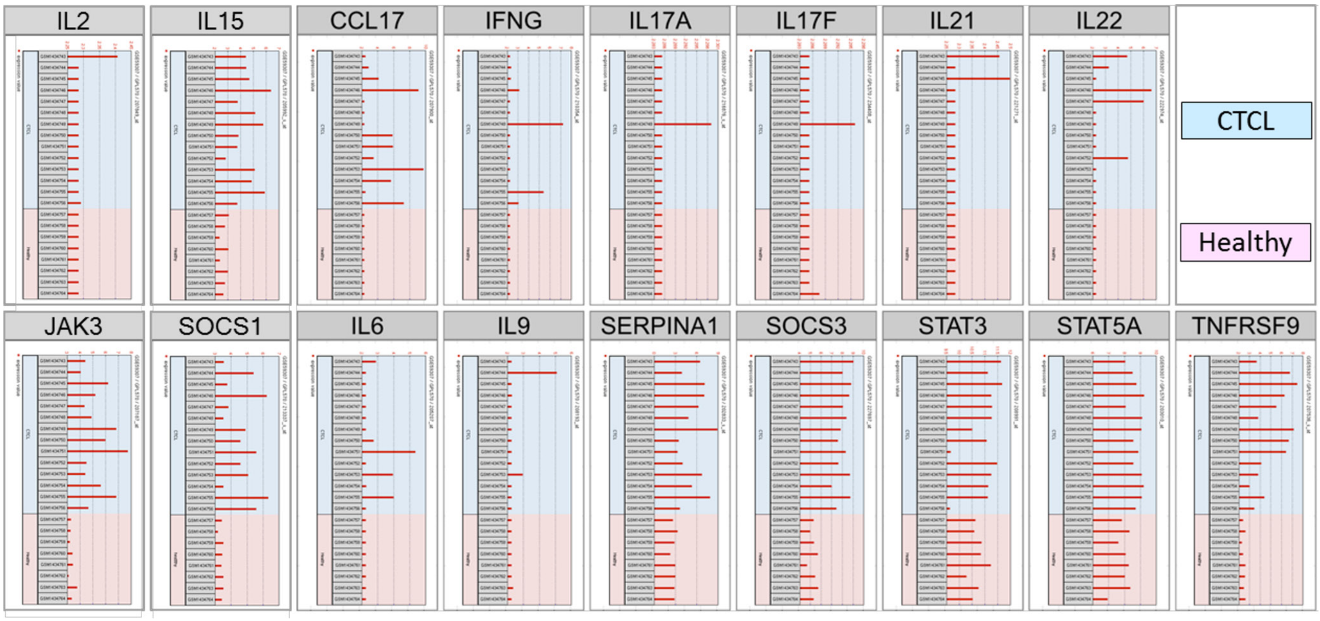

Supplementary Figure S3: Global upregulation of IL15, JAK3, SOCS1 and TNFRSF9 in CTCL patient samples. GEO microarray gene expression profiles comparing primary material from CTCL patient samples to healthy controls [28]. The GEO dataset GSE59307 was analyzed using the supplemented online analysis tools. Diagrams show the expression of genes that are subject of IL-2 mediated upregulation in MAC-1 cells and associated with aggressive phase (MAC-2A) expression profiles. Note conspicuous upregulation of IL15, JAK3, SOCS1 and TNFRSF9 in all or CCL17, IFNG, IL17A/F, IL21, IL22, IL6 and IL9 upregulation in some patient samples.

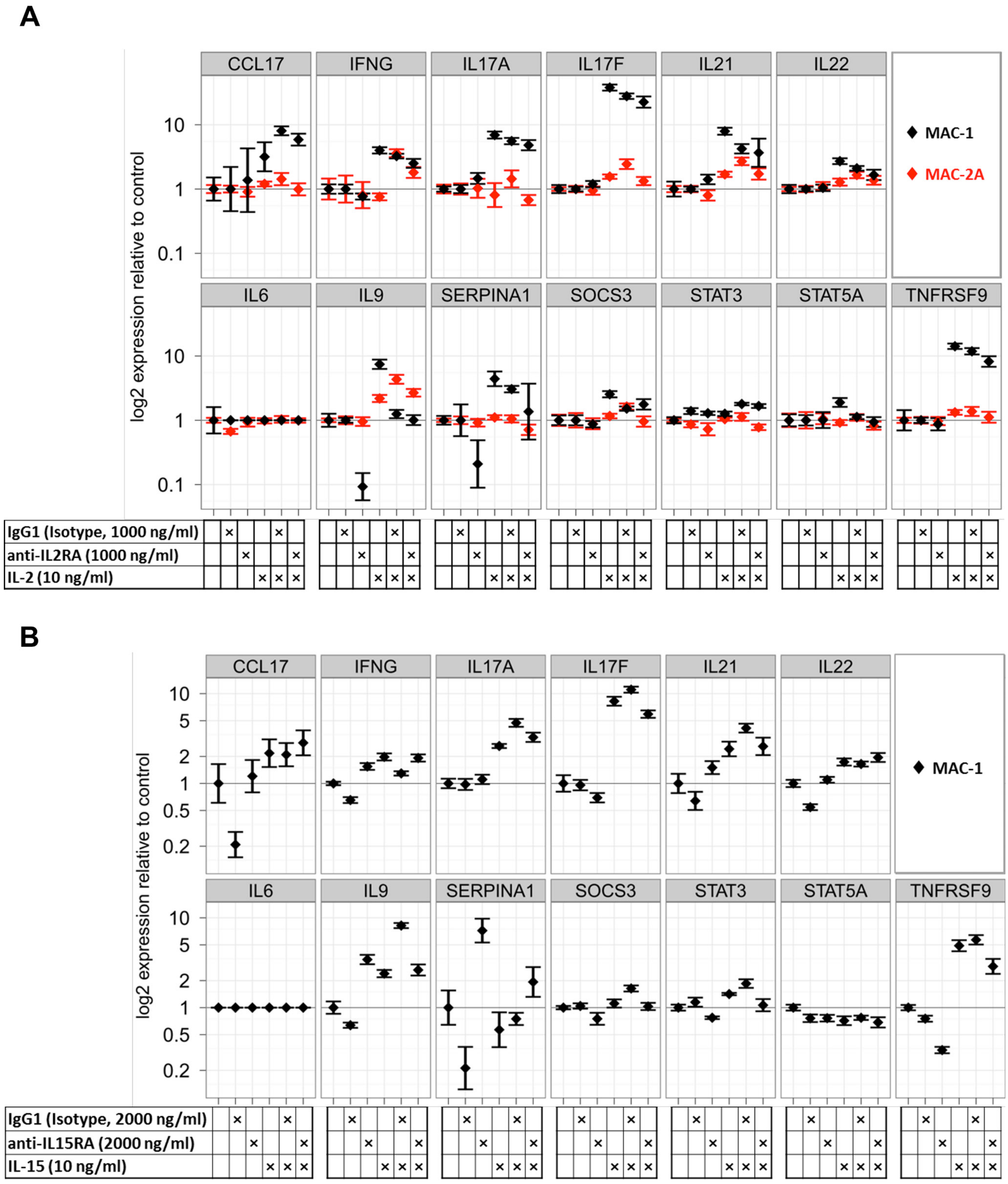

Supplementary Figure S4: IL-2 and IL-15 receptor driven gene induction. The effect of antibody mediated IL-2 (**A**) and IL-15 (**B**) receptor blocking on the inductive effect of IL-2 is shown relative to vehicle (PBS) and isotype controls. Cells were treated with every combination of the indicated factors for 72 h. Note the greater sensitivity of receptor blockade in MAC-1 cells both for IL-2 and IL-15 signaling.

Supplementary Table S1: Whole exome sequencing mutations

Chr	Pos	Variant	Gene	MAC-1	MAC-2A
1	104117852	Var_1_104117852	AMY2B	G – > A	---
1	107979368	Var_1_107979368	NTNG1	G – > A	---
1	109794593	Var_1_109794593	CELSR2	C – > T	C – > T
1	109953670	Var_1_109953670	PSMA5	T – > A	---
1	110655337	Var_1_110655337	UBL4B	A – > G	A – > G
1	12089852	Var_1_12089852	MIIP	---	T – > A
1	12175658	Var_1_12175658	TNFRSF8 (CD30)	G – > A	G – > A
1	12921104	Var_1_12921104	PRAMEF2	---	G – > A
1	14109084	Var_1_14109084	PRDM2	T – > G	T – > G
1	144866643	Var_1_144866643	PDE4DIP	G – > A	G – > A
1	144871738	Var_1_144871738	PDE4DIP	C – > A	C – > A
1	144871755	Var_1_144871755	PDE4DIP	A – > T	A – > T
1	144882823	Var_1_144882823	PDE4DIP	C – > T	C – > T
1	144916748	Var_1_144916748	PDE4DIP	C – > G	C – > G
1	144918957	Var_1_144918957	PDE4DIP	T – > A	T – > A
1	144931330	Var_1_144931330	PDE4DIP	C – > T	C – > T
1	144994658	Var_1_144994658	PDE4DIP	C – > A	C – > A
1	147084815	Var_1_147084815	BCL9	C – > T	---
1	151104950	Var_1_151104950	SEMA6C	G – > A	G – > A
1	151751274	Var_1_151751274	TDRKH	C – > G	C – > G
1	152276671	Var_1_152276671	FLG	C – > A	C – > A
1	152285860	Var_1_152285860	FLG	C – > T	C – > T
1	156907115	Var_1_156907115	ARHGEF11	T – > C	T – > C
1	157504592	Var_1_157504592	FCRL5	---	C – > T
1	160849144	Var_1_160849144	ITLN1	---	---
1	161141863	Var_1_161141863	B4GALT3	---	G – > A
1	16342125	Var_1_16342125	HSPB7	---	G – > A
1	16383682	Var_1_16383682	CLCNKB	G – > C	G – > C
1	17084968	Var_1_17084968	MST1P9	G – > C	G – > C
1	17266536	Var_1_17266536	CROCC	---	G – > C
1	173703337	Var_1_173703337	KLHL20	G – > T	---
1	174973879	Var_1_174973879	CACYBP	C – > G	---
1	175335160	Var_1_175335160	TNR	G – > A	---
1	17567203	Var_1_17567203	PADI1	---	C – > T
1	176809385	Var_1_176809385	PAPPA2	C – > T	---
1	180063357	Var_1_180063357	CEP350	A – > G	---
1	181762883	Var_1_181762883	CACNA1E	---	G – > A
1	183085720	Var_1_183085720	LAMC1	C – > G	---
1	185880824	Var_1_185880824	HMCN1	G – > T	---
1	19596124	Var_1_19596124	AKR7L	---	C – > T
1	196709816	Var_1_196709816	CFH	G – > T	G – > T
1	203012605	Var_1_203012605	PPFIA4	A – > T	---
1	203054651	Var_1_203054651	MYOG	G – > A	G – > A

1	205901026	Var_1_205901026	SLC26A9	C – > T	C – > T
1	20640906	Var_1_20640906	VWA5B1	C – > T	C – > T
1	223176696	Var_1_223176696	DISP1	C – > T	C – > T
1	223987656	Var_1_223987656	TP53BP2	G – > T	---
1	228471282	Var_1_228471282	OBSCN	C – > G	C – > G
1	231830171	Var_1_231830171	DISC1	C – > T	C – > T
1	236995266	Var_1_236995266	MTR	---	---
1	240492679	Var_1_240492679	FMN2	C – > G	---
1	241886645	Var_1_241886645	WDR64	G – > C	G – > C
1	243329049	Var_1_243329049	CEP170	G – > A	G – > A
1	245851609	Var_1_245851609	KIF26B	C – > G	C – > G
1	247769276	Var_1_247769276	OR2G3	T – > A	---
1	248059823	Var_1_248059823	OR2W3	G – > A	G – > A
1	248085124	Var_1_248085124	OR2T8	G – > A	---
1	248457979	Var_1_248457979	OR2T12	C – > A	C – > A
1	248487768	Var_1_248487768	OR2M7	A – > G	A – > G
1	248685333	Var_1_248685333	OR2G6	C – > T	C – > T
1	2488153	Var_1_2488153	TNFRSF14	A – > G	A – > G
1	32497224	Var_1_32497224	KHDRBS1	A – > G	A – > G
1	33985234	Var_1_33985234	CSMD2	---	T – > C
1	38260158	Var_1_38260158	MANEAL	---	T – > C
1	51873914	Var_1_51873914	EPS15	---	G – > A
1	70493884	Var_1_70493884	LRRC7	---	C – > G
1	86227102	Var_1_86227102	COL24A1	---	G – > A
1	89522646	Var_1_89522646	GBP1	G – > C	G – > C
1	915970	Var_1_915970	C1orf170	---	G – > A
1	91973831	Var_1_91973831	CDC7	C – > G	C – > G
1	94140470	Var_1_94140470	BCAR3	A – > T	A – > T
1	94471104	Var_1_94471104	ABCA4	T – > C	T – > C
1	94495011	Var_1_94495011	ABCA4	---	---
10	102771744	Var_10_102771744	PDZD7	C – > T	C – > T
10	105967573	Var_10_105967573	WDR96	C – > A	C – > A
10	106927065	Var_10_106927065	SORCS3	T – > A	---
10	118386427	Var_10_118386427	PNLIPRP2	G – > T	---
10	118424302	Var_10_118424302	C10orf82	G – > A	---
10	126477703	Var_10_126477703	METTL10	C – > T	C – > T
10	129906427	Var_10_129906427	MKI67	C – > A	C – > A
10	135278229	Var_10_135278229	RP11-108K14.4	A – > G	A – > G
10	17107605	Var_10_17107605	CUBN	G – > T	---
10	21462723	Var_10_21462723	NEBL	---	G – > A
10	22657592	Var_10_22657592	SPAG6	G – > A	---
10	30629220	Var_10_30629220	MTPAP	G – > A	---
10	46999484	Var_10_46999484	GPRIN2	G – > T	G – > T
10	48389841	Var_10_48389841	RBP3	C – > T	C – > T
10	5196358	Var_10_5196358	AKR1CL1	A – > C	---

10	55780139	Var_10_55780139	PCDH15	C – > A	---
10	60580159	Var_10_60580159	BICC1	---	G – > A
10	61819543	Var_10_61819543	ANK3	C – > A	C – > A
10	70716061	Var_10_70716061	DDX21	C – > T	C – > T
10	73544086	Var_10_73544086	CDH23	G – > A	G – > A
10	73594200	Var_10_73594200	PSAP	T – > G	---
10	79581028	Var_10_79581028	DLG5	G – > A	G – > A
10	79590510	Var_10_79590510	DLG5	C – > G	C – > G
10	82013183	Var_10_82013183	AL359195.1	C – > T	C – > T
10	82013316	Var_10_82013316	AL359195.1	A – > G	A – > G
10	91359052	Var_10_91359052	PANK1	---	---
10	93726465	Var_10_93726465	BTAF1	G – > T	---
10	95134707	Var_10_95134707	MYOF	G – > A	G – > A
10	95422918	Var_10_95422918	PDE6C	A – > G	---
11	108032707	Var_11_108032707	NPAT	G – > T	G – > T
11	108384339	Var_11_108384339	EXPH5	---	G – > A
11	110050628	Var_11_110050628	RDX	C – > T	C – > T
11	111590605	Var_11_111590605	SIK2	C – > T	C – > T
11	114577336	Var_11_114577336	FAM55B	G – > A	---
11	114577337	Var_11_114577337	FAM55B	A – > T	---
11	118352769	Var_11_118352769	MLL	G – > A	G – > A
11	121483520	Var_11_121483520	SORL1	G – > A	---
11	121483521	Var_11_121483521	SORL1	G – > A	---
11	124016058	Var_11_124016058	VWA5A	C – > T	C – > T
11	125547731	Var_11_125547731	ACRV1	---	C – > T
11	130332457	Var_11_130332457	ADAMTS15	T – > C	T – > C
11	134119138	Var_11_134119138	THYN1	G – > A	G – > A
11	18044438	Var_11_18044438	TPH1	---	G – > A
11	18102014	Var_11_18102014	SAAL1	---	---
11	1910635	Var_11_1910635	C11orf89	C – > G	C – > G
11	1911573	Var_11_1911573	C11orf89	G – > A	G – > A
11	193112	Var_11_193112	SCGB1C1	C – > T	C – > T
11	32858321	Var_11_32858321	PRRG4	G – > A	---
11	4510587	Var_11_4510587	OR52K1	C – > T	C – > T
11	4608542	Var_11_4608542	OR52I2	C – > T	C – > T
11	46396385	Var_11_46396385	DGKZ	---	C – > T
11	46396386	Var_11_46396386	DGKZ	---	C – > T
11	48346791	Var_11_48346791	OR4C3	C – > T	C – > T
11	48346962	Var_11_48346962	OR4C3	A – > G	A – > G
11	48347067	Var_11_48347067	OR4C3	C – > T	C – > T
11	48347306	Var_11_48347306	OR4C3	G – > T	G – > T
11	48387424	Var_11_48387424	OR4C5	G – > T	G – > T
11	4968117	Var_11_4968117	OR51A4	C – > T	C – > T
11	4968131	Var_11_4968131	OR51A4	A – > G	A – > G
11	55872657	Var_11_55872657	OR8H2	A – > C	A – > C

11	56020475	Var_11_56020475	OR5T3	---	C – > G
11	56468368	Var_11_56468368	OR9G1	C – > T	C – > T
11	5776626	Var_11_5776626	OR52N4	C – > T	C – > T
11	6024207	Var_11_6024207	OR56A4	T – > C	T – > C
11	62766510	Var_11_62766510	SLC22A8	---	C – > T
11	65380966	Var_11_65380966	MAP3K11	---	C – > T
11	6569267	Var_11_6569267	DNHD1	C – > T	C – > T
11	66082338	Var_11_66082338	CD248	G – > A	G – > A
11	66455707	Var_11_66455707	SPTBN2	---	---
11	66617824	Var_11_66617824	PC	T – > G	---
11	68773317	Var_11_68773317	MRGPRF	C – > G	C – > G
11	6891113	Var_11_6891113	OR10A2	A – > G	A – > G
11	7021500	Var_11_7021500	ZNF214	A – > G	A – > G
11	71726622	Var_11_71726622	NUMA1	G – > A	G – > A
11	74053547	Var_11_74053547	PGM2L1	C – > T	C – > T
11	77043864	Var_11_77043864	PAK1	---	C – > T
11	85438188	Var_11_85438188	SYTL2	C – > G	C – > G
11	86666088	Var_11_86666088	FZD4	G – > A	G – > A
11	92085969	Var_11_92085969	FAT3	---	A – > T
11	94532885	Var_11_94532885	AMOTL1	---	C – > T
12	10659228	Var_12_10659228	AC068775.1	A – > T	A – > T
12	109205090	Var_12_109205090	SSH1	A – > G	---
12	109647147	Var_12_109647147	ACACB	---	C – > T
12	109845660	Var_12_109845660	MYO1H	T – > A	T – > A
12	11183642	Var_12_11183642	TAS2R31	A – > G	A – > G
12	113376388	Var_12_113376388	OAS3	G – > A	G – > A
12	121600253	Var_12_121600253	P2RX7	T – > C	T – > C
12	12939892	Var_12_12939892	APOLD1	G – > A	G – > A
12	21733416	Var_12_21733416	GYS2	T – > C	---
12	234962	Var_12_234962	IQSEC3	---	G – > A
12	26714756	Var_12_26714756	ITPR2	C – > T	C – > T
12	301741	Var_12_301741	SLC6A12	---	C – > T
12	30882157	Var_12_30882157	CAPRIN2	G – > A	G – > A
12	31237542	Var_12_31237542	DDX11	G – > A	G – > A
12	48272895	Var_12_48272895	VDR	A – > G	A – > G
12	52201135	Var_12_52201135	SCN8A	G – > T	---
12	52761153	Var_12_52761153	KRT85	C – > A	---
12	53646726	Var_12_53646726	MFSD5	---	C – > T
12	54367690	Var_12_54367690	HOXC11	C – > T	C – > T
12	55968452	Var_12_55968452	OR2AP1	C – > G	C – > G
12	56079053	Var_12_56079053	ITGA7	G – > A	G – > A
12	56631435	Var_12_56631435	SLC39A5	---	G – > A
12	57849399	Var_12_57849399	INHBE	C – > T	C – > T
12	6127833	Var_12_6127833	VWF	T – > C	T – > C
12	65720714	Var_12_65720714	MSRB3	---	G – > A

12	69084468	Var_12_69084468	NUP107	---	---
12	71139698	Var_12_71139698	PTPRR	---	C – > T
12	80855825	Var_12_80855825	PTPRQ	C – > T	C – > T
13	24823894	Var_13_24823894	SPATA13	C – > T	C – > T
13	24890157	Var_13_24890157	C1QTNF9	C – > T	C – > T
13	26138164	Var_13_26138164	ATP8A2	---	C – > T
13	28130528	Var_13_28130528	LNX2	C – > T	C – > T
13	38151929	Var_13_38151929	POSTN	---	G – > A
13	61986320	Var_13_61986320	PCDH20	---	G – > A
13	79176674	Var_13_79176674	POU4F1	G – > A	G – > A
14	101011342	Var_14_101011342	BEGAIN	G – > A	G – > A
14	102476636	Var_14_102476636	DYNC1H1	---	C – > T
14	104640154	Var_14_104640154	KIF26A	T – > A	---
14	104644121	Var_14_104644121	KIF26A	---	G – > A
14	106845417	Var_14_106845417	IGHV3-35	C – > T	C – > T
14	106866465	Var_14_106866465	IGHV3-38	A – > G	A – > G
14	106866675	Var_14_106866675	IGHV3-38	C – > G	C – > G
14	106877837	Var_14_106877837	IGHV4-39	C – > A	C – > A
14	20249176	Var_14_20249176	OR4M1	G – > A	G – > A
14	20443943	Var_14_20443943	OR4K15	A – > G	A – > G
14	20940606	Var_14_20940606	PNP	G – > A	G – > A
14	21467928	Var_14_21467928	SLC39A2	T – > C	T – > C
14	23844979	Var_14_23844979	IL25	C – > T	C – > T
14	24911580	Var_14_24911580	SDR39U1	C – > T	---
14	44973730	Var_14_44973730	FSCB	G – > A	G – > A
14	64988974	Var_14_64988974	ZBTB1	A – > G	---
14	74975348	Var_14_74975348	LTBP2	G – > A	G – > A
14	77793207	Var_14_77793207	GSTZ1	G – > A	G – > A
14	91681784	Var_14_91681784	C14orf159	G – > A	G – > A
14	92920262	Var_14_92920262	SLC24A4	A – > G	A – > G
14	93994909	Var_14_93994909	KIAA1409	---	---
15	23014502	Var_15_23014502	NIPA2	C – > T	C – > T
15	33926871	Var_15_33926871	RYR3	---	C – > T
15	40056051	Var_15_40056051	FSIP1	---	G – > A
15	40488756	Var_15_40488756	BUB1B	---	A – > G
15	42120342	Var_15_42120342	JMJD7	---	A – > G
15	42145083	Var_15_42145083	SPTBN5	A – > G	A – > G
15	42362967	Var_15_42362967	PLA2G4D	C – > T	C – > T
15	43893734	Var_15_43893734	STRC	G – > A	G – > A
15	44057664	Var_15_44057664	PDIA3	C – > T	---
15	44918698	Var_15_44918698	SPG11	A – > G	---
15	55724794	Var_15_55724794	DYX1C1	---	C – > T
15	62199482	Var_15_62199482	VPS13C	C – > A	---
15	63419686	Var_15_63419686	LACTB	---	A – > T
15	74290511	Var_15_74290511	PML	C – > T	---

15	75094673	Var_15_75094673	CSK	C – > T	---
15	76079229	Var_15_76079229	AC019294.1	G – > T	---
15	79045517	Var_15_79045517	AC022748.1	G – > A	G – > A
15	88726698	Var_15_88726698	NTRK3	G – > A	G – > A
16	11349023	Var_16_11349023	SOCS1	---	C – > T
16	11349104	Var_16_11349104	SOCS1	---	C – > T
16	13297392	Var_16_13297392	SHISA9	---	C – > T
16	20441053	Var_16_20441053	ACSM5	C – > G	C – > G
16	21210893	Var_16_21210893	ZP2	---	C – > T
16	23768594	Var_16_23768594	AC130454.2	---	G – > A
16	27761387	Var_16_27761387	KIAA0556	G – > A	G – > A
16	28603655	Var_16_28603655	SULT1A2	T – > G	T – > G
16	29908385	Var_16_29908385	SEZ6L2	---	G – > A
16	3119304	Var_16_3119304	IL32	A – > G	A – > G
16	3165489	Var_16_3165489	ZNF205	A – > T	A – > T
16	3789597	Var_16_3789597	CREBBP	---	C – > A
16	48155679	Var_16_48155679	ABCC12	---	G – > A
16	5046429	Var_16_5046429	SEC14L5	---	C – > T
16	50733465	Var_16_50733465	NOD2	C – > T	---
16	56921849	Var_16_56921849	SLC12A3	G – > A	---
16	71319237	Var_16_71319237	FTSJD1	G – > A	G – > A
16	74382834	Var_16_74382834	AC009053.1	T – > C	T – > C
16	74425569	Var_16_74425569	NPIPL2	C – > T	C – > T
16	75579264	Var_16_75579264	TMEM231	C – > A	---
16	81209247	Var_16_81209247	PKD1L2	C – > T	C – > T
16	819439	Var_16_819439	MSLNL	---	C – > T
16	84872160	Var_16_84872160	CRISPLD2	---	C – > T
16	87925430	Var_16_87925430	CA5A	---	T – > C
16	89777123	Var_16_89777123	C16orf7	C – > T	C – > T
17	10215896	Var_17_10215896	MYH13	---	C – > T
17	10298599	Var_17_10298599	MYH8	C – > T	C – > T
17	1173904	Var_17_1173904	BHLHA9	C – > A	---
17	15510888	Var_17_15510888	RP11-385D13.1	G – > A	G – > A
17	15510988	Var_17_15510988	RP11-385D13.1	T – > C	T – > C
17	17696756	Var_17_17696756	RAI1	---	C – > T
17	17997274	Var_17_17997274	DRG2	---	C – > T
17	21203893	Var_17_21203893	MAP2K3	T – > C	T – > C
17	21318782	Var_17_21318782	KCNJ12	G – > A	G – > A
17	21319069	Var_17_21319069	KCNJ12	G – > A	G – > A
17	21319087	Var_17_21319087	KCNJ12	G – > A	G – > A
17	21319121	Var_17_21319121	KCNJ12	C – > T	C – > T
17	21319208	Var_17_21319208	KCNJ12	C – > T	C – > T
17	21319230	Var_17_21319230	KCNJ12	G – > C	G – > C
17	21319943	Var_17_21319943	KCNJ12	A – > G	A – > G
17	26961753	Var_17_26961753	KIAA0100	C – > T	C – > T

17	30980891	Var_17_30980891	MYO1D	---	C – > T
17	3119068	Var_17_3119068	OR1A1	G – > A	---
17	34072264	Var_17_34072264	GAS2L2	G – > T	G – > T
17	37792096	Var_17_37792096	PPP1R1B	---	C – > A
17	38319915	Var_17_38319915	CASC3	C – > T	C – > T
17	39114962	Var_17_39114962	KRT39	C – > T	C – > T
17	39134528	Var_17_39134528	KRT40	G – > A	G – > A
17	39137154	Var_17_39137154	KRT40	C – > G	C – > G
17	39137387	Var_17_39137387	KRT40	C – > T	C – > T
17	39139370	Var_17_39139370	KRT40	G – > A	G – > A
17	39156027	Var_17_39156027	KRTAP3-2	G – > A	G – > A
17	39525750	Var_17_39525750	KRT33B	C – > T	C – > T
17	39728000	Var_17_39728000	KRT9	C – > T	C – > T
17	39967770	Var_17_39967770	LEPREL4	C – > T	C – > T
17	40946808	Var_17_40946808	WNK4	C – > A	C – > A
17	43922586	Var_17_43922586	AC217771.1	C – > T	C – > T
17	45774134	Var_17_45774134	TBKBP1	---	G – > A
17	4579389	Var_17_4579389	AC091153.1	---	G – > A
17	46894377	Var_17_46894377	TTLL6	A – > C	A – > C
17	4794812	Var_17_4794812	MINK1	C – > T	C – > T
17	4855201	Var_17_4855201	ENO3	C – > A	C – > A
17	5084946	Var_17_5084946	ZNF594	C – > T	---
17	61987570	Var_17_61987570	GH1	---	G – > T
17	62950501	Var_17_62950501	AC037487.1	T – > A	T – > A
17	63221214	Var_17_63221214	RGS9	G – > A	G – > A
17	6908625	Var_17_6908625	ALOX12	---	G – > A
17	72439962	Var_17_72439962	GPRC5C	A – > G	A – > G
17	73747142	Var_17_73747142	ITGB4	---	G – > A
17	74470523	Var_17_74470523	RHBDF2	G – > A	G – > A
17	76130987	Var_17_76130987	TMC8	G – > T	G – > T
17	77082333	Var_17_77082333	ENGASE	C – > T	C – > T
17	77099331	Var_17_77099331	RBFOX3	A – > C	---
17	80065395	Var_17_80065395	CCDC57	---	---
17	80439004	Var_17_80439004	NARF	A – > G	A – > G
17	915154	Var_17_915154	ABR	T – > C	---
18	18964319	Var_18_18964319	GREB1L	---	C – > T
18	25573559	Var_18_25573559	CDH2	C – > T	---
18	31319757	Var_18_31319757	ASXL3	---	C – > T
18	33840016	Var_18_33840016	MOCOS	G – > A	G – > A
18	43495546	Var_18_43495546	EPG5	---	G – > A
18	59212371	Var_18_59212371	CDH20	---	---
18	60383982	Var_18_60383982	PHLPP1	---	G – > A
18	74091629	Var_18_74091629	ZNF516	---	C – > T
18	9887429	Var_18_9887429	TXNDC2	C – > T	C – > T
19	1045173	Var_19_1045173	ABCA7	G – > C	G – > C

19	12243995	Var_19_12243995	ZNF20	C – > G	C – > G
19	13006916	Var_19_13006916	GCDH	C – > T	---
19	1397443	Var_19_1397443	GAMT	G – > A	G – > A
19	14815918	Var_19_14815918	ZNF333	A – > C	A – > C
19	16437696	Var_19_16437696	KLF2	G – > A	G – > A
19	17170885	Var_19_17170885	HAUS8	C – > T	C – > T
19	17945696	Var_19_17945696	JAK3	C – > T	C – > T
19	20727463	Var_19_20727463	ZNF737	T – > C	T – > C
19	2403185	Var_19_2403185	TMPRSS9	C – > T	C – > T
19	3478916	Var_19_3478916	C19orf77	G – > C	G – > C
19	35434648	Var_19_35434648	ZNF30	T – > C	T – > C
19	35448919	Var_19_35448919	ZNF792	C – > T	C – > T
19	35449315	Var_19_35449315	ZNF792	---	C – > T
19	36365736	Var_19_36365736	APLP1	G – > A	G – > A
19	38875072	Var_19_38875072	GGN	G – > C	G – > C
19	40329802	Var_19_40329802	FBL	C – > T	C – > T
19	40408821	Var_19_40408821	FCGBP	C – > G	C – > G
19	40904547	Var_19_40904547	PRX	G – > A	G – > A
19	41382543	Var_19_41382543	CYP2A7	C – > T	C – > T
19	41383799	Var_19_41383799	CYP2A7	G – > A	G – > A
19	41386136	Var_19_41386136	CYP2A7	A – > C	A – > C
19	4174694	Var_19_4174694	SIRT6	---	T – > G
19	4267681	Var_19_4267681	CCDC94	C – > T	C – > T
19	44235810	Var_19_44235810	SMG9	G – > A	G – > A
19	45491354	Var_19_45491354	CLPTM1	C – > T	---
19	49106995	Var_19_49106995	FAM83E	G – > A	G – > A
19	49793947	Var_19_49793947	SLC6A16	G – > A	---
19	50334047	Var_19_50334047	MED25	C – > T	C – > T
19	50985170	Var_19_50985170	C19orf63	C – > T	C – > T
19	51584867	Var_19_51584867	KLK14	C – > T	C – > T
19	51768733	Var_19_51768733	C19orf75	C – > T	---
19	51850290	Var_19_51850290	ETFB	G – > A	G – > A
19	53304563	Var_19_53304563	ZNF28	T – > C	T – > C
19	53572435	Var_19_53572435	ZNF160	---	T – > G
19	53856349	Var_19_53856349	ZNF845	A – > T	A – > T
19	55179364	Var_19_55179364	LILRB4	A – > G	A – > G
19	55870260	Var_19_55870260	FAM71E2	G – > A	G – > A
19	56720393	Var_19_56720393	ZSCAN5C	C – > T	C – > T
19	5720074	Var_19_5720074	LONP1	G – > T	G – > T
19	57839690	Var_19_57839690	ZNF543	A – > T	A – > T
19	57956195	Var_19_57956195	ZNF749	---	---
19	58048770	Var_19_58048770	ZNF549	C – > T	C – > T
19	5831840	Var_19_5831840	FUT6	C – > T	C – > T
19	58420699	Var_19_58420699	ZNF417	C – > A	C – > A
19	5843784	Var_19_5843784	FUT3	A – > T	---

19	58601694	Var_19_58601694	ZSCAN18	---	G – > A
19	58982044	Var_19_58982044	ZNF324	C – > T	C – > T
19	59012675	Var_19_59012675	SLC27A5	C – > T	C – > T
2	112843653	Var_2_112843653	TMEM87B	A – > C	A – > C
2	121742108	Var_2_121742108	GLI2	G – > T	---
2	131521887	Var_2_131521887	FAM123C	C – > T	C – > T
2	141115619	Var_2_141115619	LRP1B	A – > T	---
2	141116420	Var_2_141116420	LRP1B	C – > T	C – > T
2	154334856	Var_2_154334856	RPRM	A – > G	A – > G
2	163128828	Var_2_163128828	IFIH1	C – > T	C – > T
2	186697847	Var_2_186697847	FSIP2	---	G – > A
2	18766166	Var_2_18766166	NT5C1B-RDH14	---	---
2	196602662	Var_2_196602662	DNAH7	A – > G	A – > G
2	198359465	Var_2_198359465	HSPD1	---	---
2	209033138	Var_2_209033138	C2orf80	C – > T	C – > T
2	210752840	Var_2_210752840	UNC80	C – > T	---
2	214012516	Var_2_214012516	IKZF2	---	C – > T
2	222433442	Var_2_222433442	EPHA4	---	C – > T
2	222433443	Var_2_222433443	EPHA4	---	C – > T
2	227945185	Var_2_227945185	COL4A4	C – > T	---
2	228159760	Var_2_228159760	COL4A3	---	G – > A
2	233697657	Var_2_233697657	GIGYF2	A – > G	A – > G
2	237123093	Var_2_237123093	ASB18	---	G – > A
2	237395487	Var_2_237395487	IQCA1	---	C – > T
2	240984789	Var_2_240984789	OR6B3	C – > T	C – > T
2	241534561	Var_2_241534561	CAPN10	---	A – > G
2	27121575	Var_2_27121575	DPYSL5	---	C – > T
2	27746289	Var_2_27746289	GCKR	G – > C	G – > C
2	30964808	Var_2_30964808	CAPN13	---	G – > A
2	55561952	Var_2_55561952	CCDC88A	---	C – > T
2	71591181	Var_2_71591181	ZNF638	---	C – > T
2	73717921	Var_2_73717921	ALMS1	C – > G	C – > G
2	74642919	Var_2_74642919	C2orf81	A – > C	---
2	74750642	Var_2_74750642	DQX1	---	G – > A
2	89309694	Var_2_89309694	IGKV1-9	G – > A	G – > A
2	96604591	Var_2_96604591	AC073995.2	A – > G	A – > G
2	96619734	Var_2_96619734	AC073995.2	C – > T	C – > T
2	97877478	Var_2_97877478	ANKRD36	G – > A	G – > A
20	2097362	Var_20_2097362	STK35	---	---
20	2597872	Var_20_2597872	TMC2	C – > A	---
20	3180666	Var_20_3180666	DDRGK1	C – > A	C – > A
20	33000329	Var_20_33000329	ITCH	C – > T	---
20	33148772	Var_20_33148772	PIGU	---	---
20	33575434	Var_20_33575434	MYH7B	C – > T	C – > T
20	3673351	Var_20_3673351	SIGLEC1	---	C – > T

20	39981502	Var_20_39981502	LPIN3	C – > A	C – > A
20	44596262	Var_20_44596262	ZNF335	C – > T	C – > T
20	45700844	Var_20_45700844	EYA2	---	G – > A
20	47247325	Var_20_47247325	PREX1	A – > T	A – > T
20	49621307	Var_20_49621307	KCNG1	C – > T	---
20	50401135	Var_20_50401135	SALL4	G – > A	---
20	55803463	Var_20_55803463	BMP7	---	C – > T
20	60768573	Var_20_60768573	GTPBP5	C – > T	C – > T
20	60907761	Var_20_60907761	LAMA5	G – > A	G – > A
20	60927318	Var_20_60927318	LAMA5	C – > T	---
20	61303274	Var_20_61303274	SLCO4A1	G – > A	G – > A
20	62161517	Var_20_62161517	PTK6	C – > T	---
21	15561525	Var_21_15561525	LIPI	---	A – > T
21	31797957	Var_21_31797957	KRTAP13-3	A – > G	---
21	34922157	Var_21_34922157	SON	C – > T	C – > T
21	39755768	Var_21_39755768	ERG	C – > T	---
21	44171299	Var_21_44171299	PDE9A	T – > C	---
21	45535177	Var_21_45535177	PWP2	C – > T	C – > T
21	45959918	Var_21_45959918	KRTAP10-1	G – > A	---
21	45970920	Var_21_45970920	KRTAP10-2	C – > T	C – > T
21	46011468	Var_21_46011468	KRTAP10-6	G – > A	G – > A
21	46047779	Var_21_46047779	KRTAP10-9	G – > A	G – > A
22	17468918	Var_22_17468918	GAB4	C – > A	---
22	17640045	Var_22_17640045	CECR5	---	G – > A
22	18304804	Var_22_18304804	XXbac-B461K10.4	T – > A	---
22	18905964	Var_22_18905964	PRODH	C – > T	---
22	21988438	Var_22_21988438	CCDC116	C – > T	C – > T
22	22042377	Var_22_22042377	PPIL2	C – > A	C – > A
22	24574054	Var_22_24574054	CABIN1	G – > A	G – > A
22	24579157	Var_22_24579157	SUSD2	G – > T	G – > T
22	28208729	Var_22_28208729	PITPNB	A – > G	---
22	28385884	Var_22_28385884	TTC28	---	C – > T
22	29837537	Var_22_29837537	RFPL1	T – > C	T – > C
22	30197031	Var_22_30197031	ASCC2	T – > C	T – > C
22	32827391	Var_22_32827391	BPIFC	G – > A	G – > A
22	38074546	Var_22_38074546	LGALS1	G – > T	---
22	38245427	Var_22_38245427	EIF3L	T – > A	---
22	50967642	Var_22_50967642	TYMP	C – > T	---
3	10975833	Var_3_10975833	SLC6A11	T – > A	---
3	111686531	Var_3_111686531	PHLDB2	G – > A	---
3	121410949	Var_3_121410949	GOLGB1	---	---
3	121631912	Var_3_121631912	SLC15A2	G – > A	G – > A
3	13401970	Var_3_13401970	NUP210	---	C – > A
3	142443570	Var_3_142443570	TRPC1	A – > C	---
3	151166525	Var_3_151166525	IGSF10	---	A – > G

3	158315912	Var_3_158315912	MLF1	C – > T	C – > T
3	158450169	Var_3_158450169	RARRES1	C – > G	C – > G
3	171417570	Var_3_171417570	PLD1	G – > A	G – > A
3	179424788	Var_3_179424788	USP13	T – > C	T – > C
3	180693937	Var_3_180693937	FXR1	C – > T	---
3	186437936	Var_3_186437936	KNG1	---	G – > A
3	187416719	Var_3_187416719	RTP2	T – > C	T – > C
3	195452951	Var_3_195452951	MUC20	G – > C	G – > C
3	195453243	Var_3_195453243	MUC20	C – > T	C – > T
3	19921206	Var_3_19921206	EFHB	---	C – > T
3	35731608	Var_3_35731608	ARPP21	---	---
3	39140352	Var_3_39140352	GORASP1	C – > A	C – > A
3	42700436	Var_3_42700436	ZBTB47	---	G – > T
3	4358476	Var_3_4358476	SETMAR	C – > T	C – > T
3	44776005	Var_3_44776005	ZNF501	T – > G	T – > G
3	45267321	Var_3_45267321	TMEM158	---	C – > T
3	48465206	Var_3_48465206	PLXNB1	C – > T	C – > T
3	49148473	Var_3_49148473	USP19	---	G – > A
3	53260819	Var_3_53260819	TKT	C – > T	C – > T
3	55003857	Var_3_55003857	CACNA2D3	A – > C	A – > C
3	64132665	Var_3_64132665	PRICKLE2	G – > T	---
3	77671381	Var_3_77671381	ROBO2	C – > A	---
3	8574462	Var_3_8574462	LMCD1	T – > C	T – > C
3	97983711	Var_3_97983711	OR5H6	T – > C	T – > C
4	100263965	Var_4_100263965	ADH1C	C – > T	C – > T
4	104640340	Var_4_104640340	TACR3	---	G – > A
4	119947816	Var_4_119947816	SYNPO2	G – > A	G – > A
4	120166507	Var_4_120166507	USP53	---	G – > T
4	120241902	Var_4_120241902	FABP2	T – > C	---
4	123167847	Var_4_123167847	KIAA1109	T – > C	T – > C
4	126237751	Var_4_126237751	FAT4	G – > C	G – > C
4	134073828	Var_4_134073828	PCDH10	A – > T	---
4	144620679	Var_4_144620679	FREM3	---	C – > T
4	149075780	Var_4_149075780	NR3C2	---	C – > T
4	153247289	Var_4_153247289	FBXW7	---	G – > A
4	158281129	Var_4_158281129	GRIA2	---	G – > A
4	162459414	Var_4_162459414	FSTL5	---	C – > T
4	164054373	Var_4_164054373	NAF1	T – > G	T – > G
4	1843342	Var_4_1843342	LETM1	T – > C	T – > C
4	187628419	Var_4_187628419	FAT1	C – > T	C – > T
4	22436998	Var_4_22436998	GPR125	---	A – > T
4	25671464	Var_4_25671464	SLC34A2	---	G – > T
4	26431577	Var_4_26431577	RBPJ	A – > G	---
4	36126568	Var_4_36126568	ARAP2	C – > A	C – > A
4	38800315	Var_4_38800315	TLR1	T – > A	---

4	42403431	Var_4_42403431	SHISA3	T – > A	T – > A
4	493135	Var_4_493135	PIGG	G – > C	G – > C
4	54362332	Var_4_54362332	LNX1	T – > C	T – > C
4	6302816	Var_4_6302816	WFS1	---	C – > G
4	69313240	Var_4_69313240	TMPRSS11E	---	C – > T
4	74280823	Var_4_74280823	ALB	A – > G	---
4	76529113	Var_4_76529113	CDKL2	A – > G	---
4	94750364	Var_4_94750364	ATOH1	---	G – > T
5	101575078	Var_5_101575078	SLCO4C1	---	G – > A
5	102342709	Var_5_102342709	PAM	---	G – > A
5	10433728	Var_5_10433728	MARCH6	G – > A	---
5	110436345	Var_5_110436345	WDR36	A – > C	A – > C
5	118969777	Var_5_118969777	FAM170A	A – > G	A – > G
5	127609652	Var_5_127609652	FBN2	C – > T	---
5	129241056	Var_5_129241056	CHSY3	---	G – > A
5	132918994	Var_5_132918994	FSTL4	---	---
5	135692993	Var_5_135692993	TRPC7	---	C – > T
5	139751194	Var_5_139751194	SLC4A9	---	C – > G
5	140558628	Var_5_140558628	PCDHB8	T – > C	T – > C
5	141053235	Var_5_141053235	ARAP3	G – > A	---
5	141242812	Var_5_141242812	PCDH1	---	T – > A
5	143853624	Var_5_143853624	KCTD16	C – > T	---
5	145631334	Var_5_145631334	RBM27	C – > T	C – > T
5	147870386	Var_5_147870386	HTR4	G – > A	G – > A
5	149323939	Var_5_149323939	PDE6A	G – > A	G – > A
5	149576739	Var_5_149576739	SLC6A7	A – > C	---
5	157078924	Var_5_157078924	SOX30	A – > G	A – > G
5	161128694	Var_5_161128694	GABRA6	C – > G	C – > G
5	167420038	Var_5_167420038	ODZ2	C – > T	C – > T
5	170736478	Var_5_170736478	TLX3	G – > T	---
5	172113227	Var_5_172113227	NEURL1B	---	---
5	178414409	Var_5_178414409	GRM6	C – > T	C – > T
5	179306086	Var_5_179306086	TBC1D9B	G – > A	---
5	38407225	Var_5_38407225	EGFLAM	A – > C	---
5	41176678	Var_5_41176678	C6	C – > T	---
5	61688030	Var_5_61688030	DIMT1L	---	---
5	64314062	Var_5_64314062	CWC27	---	---
5	716953	Var_5_716953	ZDHHC11B	C – > T	C – > T
5	72286545	Var_5_72286545	FCHO2	G – > A	---
5	73069793	Var_5_73069793	RP11-428C6.1	---	G – > A
5	767033	Var_5_767033	ZDHHC11B	A – > C	A – > C
5	78181477	Var_5_78181477	ARSB	C – > T	C – > T
5	79025847	Var_5_79025847	CMYA5	A – > G	A – > G
5	79028819	Var_5_79028819	CMYA5	T – > G	T – > G
5	82406931	Var_5_82406931	XRCC4	T – > C	T – > C

5	82841406	Var_5_82841406	VCAN	C – > T	---
5	82841412	Var_5_82841412	VCAN	---	G – > A
5	90046509	Var_5_90046509	GPR98	G – > A	---
6	12163739	Var_6_12163739	HIVEP1	T – > C	T – > C
6	121768838	Var_6_121768838	GJA1	C – > T	C – > T
6	131215568	Var_6_131215568	EPB41L2	T – > C	---
6	1312998	Var_6_1312998	FOXQ1	T – > C	T – > C
6	137815120	Var_6_137815120	OLIG3	G – > A	G – > A
6	147527173	Var_6_147527173	STXBP5	---	---
6	160229816	Var_6_160229816	PNLDC1	---	C – > T
6	166580080	Var_6_166580080	T	---	C – > A
6	168352584	Var_6_168352584	MLLT4	G – > A	G – > A
6	21595712	Var_6_21595712	SOX4	C – > T	C – > T
6	25520501	Var_6_25520501	LRRC16A	C – > T	---
6	26124759	Var_6_26124759	HIST1H2AC	---	G – > A
6	26405816	Var_6_26405816	BTN3A1	T – > C	T – > C
6	27277212	Var_6_27277212	POM121L2	C – > A	C – > A
6	29141632	Var_6_29141632	OR2J2	T – > C	T – > C
6	29911296	Var_6_29911296	HLA-A	G – > A	G – > A
6	29911306	Var_6_29911306	HLA-A	C – > T	C – > T
6	30918400	Var_6_30918400	DPCR1	A – > G	A – > G
6	31378864	Var_6_31378864	MICA	A – > G	A – > G
6	31515994	Var_6_31515994	NFKBIL1	---	---
6	32084259	Var_6_32084259	ATF6B	T – > C	T – > C
6	32497961	Var_6_32497961	HLA-DRB5	T – > G	---
6	32497962	Var_6_32497962	HLA-DRB5	T – > C	---
6	32606722	Var_6_32606722	HLA-DQA1	A – > G	---
6	33281504	Var_6_33281504	TAPBP	C – > A	C – > A
6	36172425	Var_6_36172425	BRPF3	T – > A	T – > A
6	36343714	Var_6_36343714	ETV7	C – > T	C – > T
6	39159379	Var_6_39159379	KCNK5	G – > A	G – > A
6	40996173	Var_6_40996173	UNC5CL	G – > C	G – > C
6	41903782	Var_6_41903782	CCND3	A – > C	A – > C
6	44233216	Var_6_44233216	NFKBIE	G – > C	G – > C
6	46846043	Var_6_46846043	GPR116	T – > A	T – > A
6	46856227	Var_6_46856227	GPR116	G – > A	G – > A
6	49814379	Var_6_49814379	CRISP1	T – > C	T – > C
6	5086206	Var_6_5086206	PPP1R3G	---	C – > T
6	51890241	Var_6_51890241	PKHD1	G – > A	---
6	54002764	Var_6_54002764	MLIP	G – > A	---
6	56505081	Var_6_56505081	DST	G – > C	G – > C
6	73904899	Var_6_73904899	KCNQ5	C – > T	---
7	100841532	Var_7_100841532	MOGAT3	C – > T	C – > T
7	111379198	Var_7_111379198	DOCK4	G – > C	G – > C
7	122303308	Var_7_122303308	CADPS2	G – > A	---

7	128485240	Var_7_128485240	FLNC	C – > T	C – > T
7	141858128	Var_7_141858128	RP11-1220K2.2	A – > G	---
7	143027910	Var_7_143027910	CLCN1	G – > A	G – > A
7	143657707	Var_7_143657707	OR2F1	---	T – > C
7	147815297	Var_7_147815297	CNTNAP2	---	C – > T
7	153750096	Var_7_153750096	DPP6	---	---
7	156743095	Var_7_156743095	NOM1	G – > A	G – > A
7	21932164	Var_7_21932164	DNAH11	A – > T	---
7	2296618	Var_7_2296618	SNX8	A – > T	A – > T
7	25163578	Var_7_25163578	CYCS	T – > A	---
7	31736613	Var_7_31736613	C7orf16	T – > A	---
7	34091556	Var_7_34091556	BMPER	---	G – > A
7	45930256	Var_7_45930256	IGFBP1	C – > A	---
7	48547511	Var_7_48547511	ABCA13	C – > A	C – > A
7	55902230	Var_7_55902230	SEP T4	T – > C	T – > C
7	72395789	Var_7_72395789	POM121	C – > T	---
7	8791395	Var_7_8791395	NXPH1	G – > A	G – > A
7	940186	Var_7_940186	ADAP1	C – > A	---
7	97860597	Var_7_97860597	TECPR1	C – > T	C – > T
7	99565783	Var_7_99565783	AZGP1	C – > G	C – > G
8	101008701	Var_8_101008701	RGS22	C – > T	C – > T
8	103293586	Var_8_103293586	UBR5	G – > A	---
8	106813316	Var_8_106813316	ZFPM2	---	A – > C
8	113253954	Var_8_113253954	CSMD3	A – > G	A – > G
8	113317016	Var_8_113317016	CSMD3	---	G – > A
8	11418773	Var_8_11418773	BLK	C – > T	C – > T
8	128428751	Var_8_128428751	POU5F1B	A – > G	---
8	128428823	Var_8_128428823	POU5F1B	G – > C	---
8	13133881	Var_8_13133881	DLC1	G – > T	G – > T
8	144810138	Var_8_144810138	FAM83H	G – > A	---
8	144945191	Var_8_144945191	EPPK1	T – > C	T – > C
8	17206488	Var_8_17206488	MTMR7	G – > A	G – > A
8	17919807	Var_8_17919807	ASAH1	A – > G	A – > G
8	27293828	Var_8_27293828	PTK2B	T – > A	---
8	27373865	Var_8_27373865	EPHX2	G – > A	G – > A
8	77895667	Var_8_77895667	PEX2	A – > G	A – > G
8	87229890	Var_8_87229890	SLC7A13	G – > A	G – > A
8	96166999	Var_8_96166999	PLEKHF2	G – > T	---
9	107288965	Var_9_107288965	OR13C4	G – > A	G – > A
9	107367008	Var_9_107367008	OR13C2	T – > C	T – > C
9	111679940	Var_9_111679940	IKBKAP	T – > C	T – > C
9	114886569	Var_9_114886569	SUSD1	C – > T	C – > T
9	117169033	Var_9_117169033	DFNB31	A – > G	A – > G
9	119495741	Var_9_119495741	ASTN2	---	G – > C
9	124074740	Var_9_124074740	GSN	---	G – > A

9	125486365	Var_9_125486365	OR1L4	C – > T	C – > T
9	125486968	Var_9_125486968	OR1L4	G – > A	G – > A
9	125512575	Var_9_125512575	OR1L6	G – > A	G – > A
9	125512826	Var_9_125512826	OR1L6	G – > A	G – > A
9	130588091	Var_9_130588091	ENG	C – > T	C – > T
9	130928633	Var_9_130928633	CIZ1	C – > T	C – > T
9	131020400	Var_9_131020400	GOLGA2	C – > G	C – > G
9	132637027	Var_9_132637027	USP20	C – > T	C – > T
9	133047497	Var_9_133047497	RP11-88G17.6	A – > G	A – > G
9	134351730	Var_9_134351730	PRRC2B	T – > G	T – > G
9	136219396	Var_9_136219396	SURF1	T – > A	T – > A
9	136582482	Var_9_136582482	SARDH	C – > A	C – > A
9	138664762	Var_9_138664762	KCNT1	C – > T	C – > T
9	140389519	Var_9_140389519	PNPLA7	---	C – > T
9	140389520	Var_9_140389520	PNPLA7	---	C – > T
9	15307197	Var_9_15307197	TTC39B	C – > G	C – > G
9	33472272	Var_9_33472272	NOL6	T – > A	---
9	33618460	Var_9_33618460	TRBV20OR9-2	A – > T	A – > T
9	33618497	Var_9_33618497	TRBV20OR9-2	G – > A	G – > A
9	33944433	Var_9_33944433	UBAP2	---	G – > A
9	35957302	Var_9_35957302	OR2S2	G – > A	---
9	82333770	Var_9_82333770	TLE4	G – > A	G – > A
9	88938668	Var_9_88938668	ZCCHC6	G – > A	G – > A
9	96052324	Var_9_96052324	WNK2	---	T – > C
9	96061441	Var_9_96061441	WNK2	G – > A	G – > A
9	97329611	Var_9_97329611	FBP2	A – > G	A – > G
X	100743826	Var_X_100743826	ARMCX4	A – > G	A – > G
X	101437635	Var_X_101437635	RP1-158I15.2	A – > G	A – > G
X	105278361	Var_X_105278361	SERPINA7	C – > A	C – > A
X	108708552	Var_X_108708552	GUCY2F	A – > G	A – > G
X	114425400	Var_X_114425400	RBMXL3	G – > A	G – > A
X	123034511	Var_X_123034511	XIAP	A – > C	A – > C
X	128782705	Var_X_128782705	APLN	C – > A	---
X	12924826	Var_X_12924826	TLR8	A – > G	A – > G
X	136113464	Var_X_136113464	GPR101	C – > A	C – > A
X	138774209	Var_X_138774209	MCF2	---	C – > T
X	140983127	Var_X_140983127	MAGEC3	G – > A	G – > A
X	150349871	Var_X_150349871	GPR50	A – > G	A – > G
X	151869765	Var_X_151869765	MAGEA6	G – > T	G – > T
X	152751332	Var_X_152751332	U82695.9	---	G – > C
X	15306063	Var_X_15306063	ASB11	T – > C	---
X	2139186	Var_X_2139186	DHRSX	C – > T	C – > T
X	26235671	Var_X_26235671	MAGEB5	G – > A	G – > A
X	2835863	Var_X_2835863	ARSD	G – > T	---
X	2836181	Var_X_2836181	ARSD	A – > T	---

X	2836184	Var_X_2836184	ARSD	C – > T	---
X	2836211	Var_X_2836211	ARSD	A – > T	---
X	2836238	Var_X_2836238	ARSD	G – > A	---
X	3238733	Var_X_3238733	MXRA5	G – > A	G – > A
X	35821055	Var_X_35821055	MAGEB16	---	---
X	48054740	Var_X_48054740	SSX5	C – > G	C – > G
X	91518138	Var_X_91518138	PCDH11X	---	---
X	99662125	Var_X_99662125	PCDH19	G – > T	---
